# Distinct roles of two myosins in *C*. *elegans* spermatid differentiation

**DOI:** 10.1371/journal.pbio.3000211

**Published:** 2019-04-16

**Authors:** Junyan Hu, Shiya Cheng, Haibin Wang, Xin Li, Sun Liu, Mengmeng Wu, Yubing Liu, Xiaochen Wang

**Affiliations:** 1 National Laboratory of Biomacromolecules, CAS Center for Excellence in Biomacromolecules, Institute of Biophysics, Chinese Academy of Sciences, Beijing, China; 2 College of Life Sciences, University of Chinese Academy of Sciences, Beijing, China; University of Michigan, UNITED STATES

## Abstract

During spermatogenesis, interconnected haploid spermatids segregate undesired cellular contents into residual bodies (RBs) before detaching from RBs. It is unclear how this differentiation process is controlled to produce individual spermatids or motile spermatozoa. Here, we developed a live imaging system to visualize and investigate this process in *C*. *elegans*. We found that non-muscle myosin 2 (NMY-2)/myosin II drives incomplete cytokinesis to generate connected haploid spermatids, which are then polarized to segregate undesired cellular contents into RBs under the control of myosin II and myosin VI. NMY-2/myosin II extends from the pseudo-cleavage furrow formed between two haploid spermatids to the spermatid poles, thus promoting RB expansion. In the meantime, defective spermatogenesis 15 (SPE-15)/myosin VI migrates from spermatids towards the expanding RB to promote spermatid budding. Loss of myosin II or myosin VI causes distinct cytoplasm segregation defects, while loss of both myosins completely blocks RB formation. We found that the final separation of spermatids from RBs is achieved through myosin VI–mediated cytokinesis, while myosin II is dispensable at this step. SPE-15/myosin VI and F-actin form a detergent-resistant actomyosin VI ring that undergoes continuous contraction to promote membrane constriction between spermatid and RB. We further identified that RGS-GAIP-interacting protein C terminus (GIPC)-1 and GIPC-2 cooperate with myosin VI to regulate contractile ring formation and spermatid release. Our study reveals distinct roles of myosin II and myosin VI in spermatid differentiation and uncovers a novel myosin VI–mediated cytokinesis process that controls spermatid release.

## Abstract 2

Haploid spermatids segregate unwanted cellular contents into residual bodies before detaching. A study in the nematode Caenorhabditis elegans shows that residual body formation and cytoplasm segregation are executed by the coordinated activity of myosin II and myosin VI, and spermatids are detached via myosin VI-mediated cytokinesis.

## Author summary

Spermatogenesis is a conserved multistep process that involves mitotic proliferation of spermatogonia, meiotic division of spermatocytes, and differentiation of haploid spermatids into mature spermatozoa. During spermatid differentiation, spermatids shed unwanted cellular contents in the form of a residual body (RB) and detach from the RB to become mature spermatozoa. A series of cellular events occur in this process, including RB formation, cytoplasm segregation, and spermatid release, but the underlying cellular mechanisms are largely unknown. In this study, by combining live-cell imaging and genetic manipulation in an optimized in vitro culture system, we monitored and investigated *Caenorhabditis elegans* spermatid differentiation with high temporal and spatial resolutions. We found that RB formation and cytoplasm segregation are regulated by 2 actin-based molecular motors, which play distinct roles in these processes. Non-muscle Myosin 2 (NMY-2)/myosin II drives incomplete cytokinesis to generate connected haploid spermatids. NMY-2 and F-actin accumulate at the pseudo-cleavage furrow and extend towards the spermatid poles to promote RB expansion. In the meantime, defective spermatogenesis 15 (SPE-15)/myosin VI co-migrates with cortical actin from spermatids towards the expanding RB to promote spermatid budding. Cellular contents are differentially segregated into spermatids and RBs during RB expansion mediated by myosin II and myosin VI, respectively. We discovered that spermatid release is achieved by myosin VI–mediated cytokinesis and that the cargo adaptor RGS-GAIP-interacting protein C terminus (GIPC) is the key regulator of myosin VI in this process. SPE-15/myosin VI and F-actin form a detergent-resistant actomyosin VI ring to promote membrane restriction that separates spermatids from the RB.

## Introduction

Spermatogenesis is a conserved multistep process that involves mitotic proliferation of spermatogonia, meiotic division of spermatocytes, and differentiation of haploid spermatids into mature spermatozoa. In *C*. *elegans*, spermatogenesis occurs in both males and hermaphrodites. Upon entering meiosis, primary spermatocytes bud off the syncytial cytoplasmic core known as the rachis and undergo meiosis, resulting in generation of 2N secondary spermatocytes that sometimes undergo incomplete cytokinesis and remain attached [1, 2]. Each secondary spermatocyte initiates meiosis II to produce 2 connected haploid spermatids, which differentiate to shed undesired cytoplasm in the form of a residual body (RB). Spermatids inherit mitochondria, Golgi-derived fibrous-body membranous organelles (FB-MOs), and a haploid nucleus, whereas all ribosomes, nearly all the actin, and most of the tubulin are packaged into the RB [1–4]. Spermatids subsequently detach from the RB and further mature into motile spermatozoa [2–6]. The RBs are phagocytosed and degraded in gonadal sheath cells by the molecular machinery that removes apoptotic cells [7]. RB formation and cytoplasm segregation also occur in flies during spermatid individualization [8, 9]. In vertebrates, incomplete cytokinesis occurs during mitosis of spermatogonia and meiosis of spermatocytes, leading to generation of a bundle of interconnected haploid spermatids [10–12]. The spermatids transform into spermatozoa through a process called spermiogenesis, which involves multiple cellular remodeling events including selective placement of unneeded cellular contents into RBs and release of sperm from RBs [13–15]. Failure of this process affects spermatid generation and activation, causing male infertility in worms, flies, and mammals [9, 16–18]. However, little is known about how RBs form and detach from spermatids and how cytoplasm segregation is achieved.

Myosins are actin-based molecular motors that translocate along actin filaments in an ATP-dependent manner. Myosins have been implicated in various aspects of spermatogenesis, including mitosis and meiosis, spermatid differentiation, and cytoplasm segregation, consistent with the extensive actin cytoskeleton remodeling observed in these processes [19, 20]. Non-muscle myosin II is crucial for cytokinesis in both mitotic and meiotic cells [21]. Myosin VI is the only minus-end–directed motor protein on actin filaments [22]. It participates in a variety of cellular processes by transporting cargos along actin or stabilizing actin cytoskeleton structures and linking them with membranes or protein complexes [23, 24]. In either case, myosin VI is assisted by associating with cargo adaptors, which mediate targeting and dimerization of the motor [23, 25]. Myosin VI is important in *Drosophila* spermatid individualization, probably by stabilizing the actin cone [26–28]. Loss of defective spermatogenesis 15 (SPE-15), the *C*. *elegans* myosin VI, affects proper partitioning of organelles and cytoskeleton components into spermatids and RBs [16]. However, it is unclear whether and how SPE-15 directly regulates the sorting process.

In this study, we developed a live-cell imaging system to visualize spermatid differentiation in *C*. *elegans*, during which spermatids shed unneeded cellular contents into RBs and detach from RBs. Our study reveals distinct roles of myosin II and myosin VI in residual body formation and cytoplasm segregation and uncovers a novel myosin VI–mediated cytokinesis that separates spermatids from RBs.

## Results

### Live-cell labeling and imaging visualize spermatid differentiation in vitro

**Fig 1:**
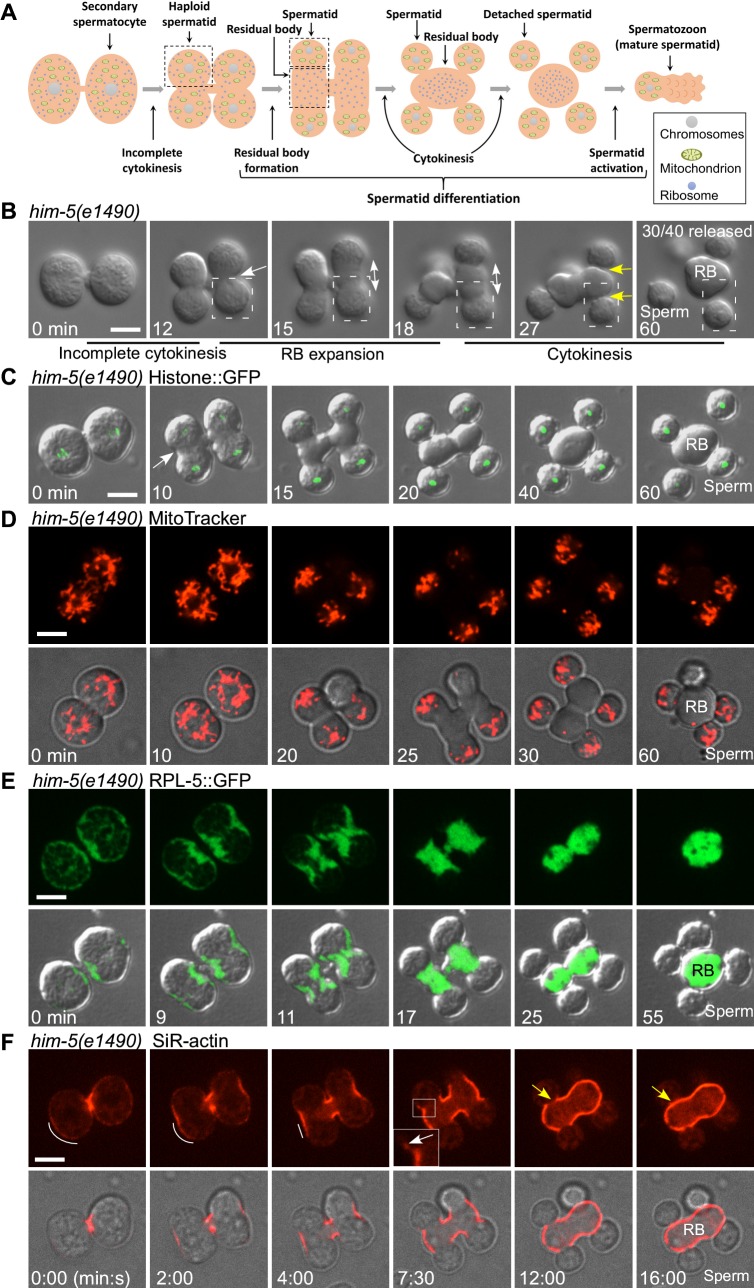
Live-cell imaging visualizes dynamics of meiosis and spermatid differentiation. (A) A diagram showing the spermatid differentiation process in *C*. *elegans*. (B) Time-lapse analysis of meiosis and differentiation in 2 connected secondary spermatocytes dissected from *him-5* males. White arrow indicates the pseudo-cleavage furrow between 2 undifferentiated spermatids. Double-headed arrow indicates RB expansion during spermatid differentiation. Yellow arrows point to cleavage furrows between spermatids and RB. Dashed line boxes indicate 1 haploid spermatid at each differentiation step. Numbers of released spermatids/total spermatids were quantified and are shown in the top right corner. (C–F) Time-lapse analysis of meiosis and differentiation in 2 connected secondary spermatocytes dissected from *him-5* males. Spermatocytes in (C) and (E) are from animals expressing Histone::GFP and RPL-5::GFP, respectively. Spermatocytes in (D) and (F) are stained by MitoTracker Red and SiR-actin, respectively. White arrow in (C) indicates the pseudo-cleavage furrow. In (F), white lines indicate the distribution and enrichment of SiR-actin signal during RB formation. The inset and white arrow show an enlarged view of a moving SiR-actin punctum with a magnification of 4×, and yellow arrows indicate the closure of SiR-actin at the scission site. Scale bars: 5 μm. GFP, green fluorescent protein; RB, residual body; RPL-5, ribosomal protein 5, large subunit; SiR, silicon-rhodamine.

**Fig 2:**
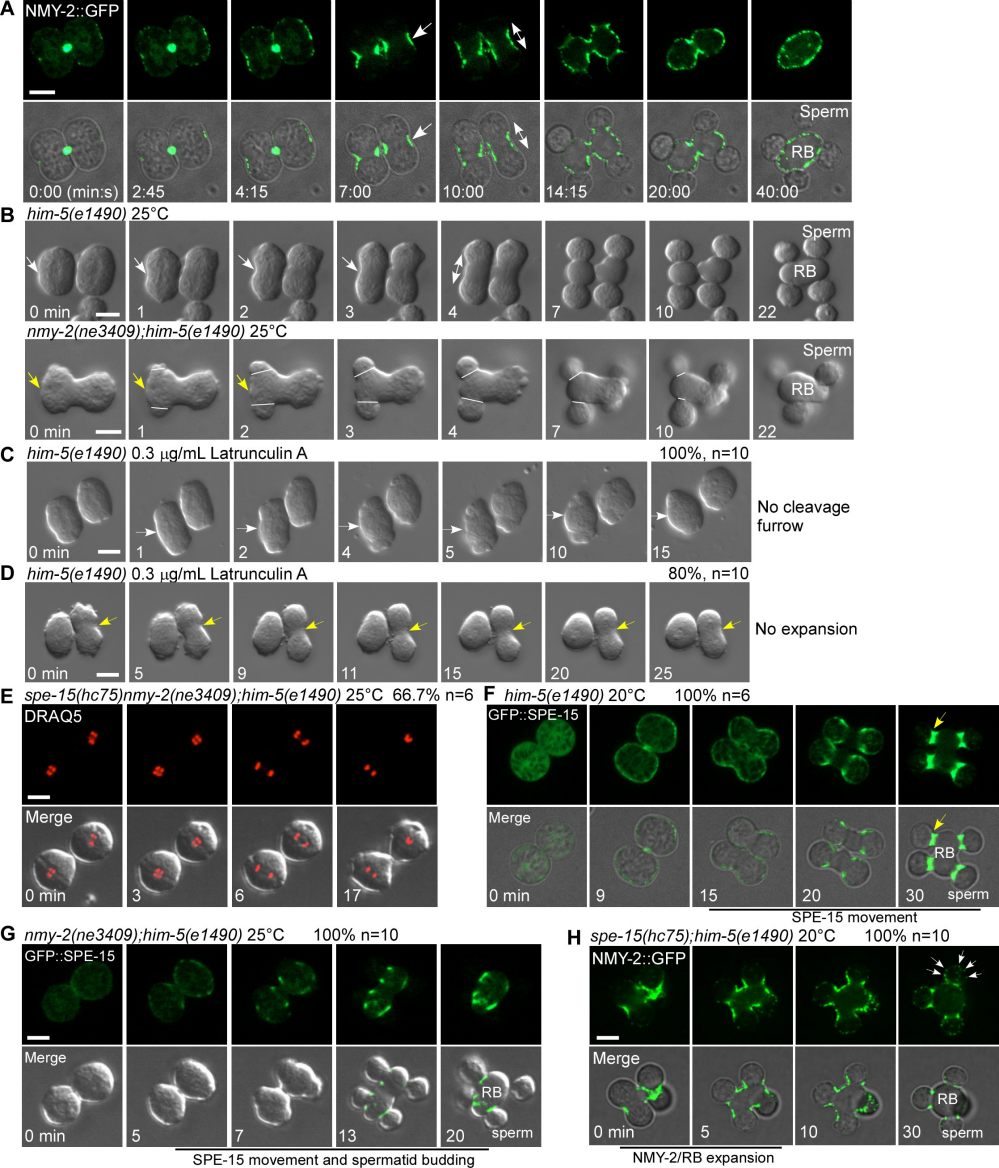
Myosin II and myosin VI play distinct roles to regulate spermatid and RB formation. (A) Time-lapse analysis of meiosis and differentiation in 2 connected secondary spermatocytes dissected from *him-5* males expressing NMY-2::GFP. Arrow indicates accumulation of NMY-2 at the pseudo-cleavage furrow between 2 undifferentiated spermatids. Double-headed arrow indicates expansion of RB and NMY-2 during spermatid differentiation. (B) Time-lapse analysis of meiosis and differentiation in secondary spermatocytes dissected from *him-5* or *nmy-2;him-5* males at the nonpermissive temperature of 25°C. White arrows indicate pseudo-cleavage furrow ingression in the middle of the dividing *him-5* secondary spermatocyte. Double-headed arrow indicates RB expansion in *him-5*. Yellow arrows point to the midpoint of the dividing secondary spermatocyte in *nmy-2;him-5* animals. White lines mark the sperm–RB boundaries and indicate the sites of spermatid budding from the remaining cell body in *nmy-2;him-5* animals. (C and D) Time-lapse images of secondary spermatocytes from *him-5* males at different developmental stages in the presence of Latrunculin A. White arrows point to the midpoint of a secondary spermatocyte. Yellow arrows indicate the pseudo-cleavage furrow between 2 undifferentiated spermatids. (E) Time-lapse images of 2 connected secondary spermatocytes dissected from *spe-15(hc75) nmy-2(ne3409);him-5(e1490)* males stained by the DNA label DRAQ5 at the nonpermissive temperature of 25°C. The DRAQ5-labeled chromosomes were segregated, but differentiation of haploid spermatids was blocked. (F) Time-lapse images of meiosis and differentiation in 2 connected secondary spermatocytes dissected from *him-5* males expressing GFP::SPE-15. Yellow arrows indicate accumulation of SPE-15 at the spermatid–RB boundary. (G) Time-lapse analysis of meiosis and differentiation in 2 connected secondary spermatocytes dissected from *nmy-2(ne3409);him-5(e1490)* males expressing GFP::SPE-15 at the nonpermissive temperature (25°C). (H) Time-lapse analysis of differentiation in connected spermatids dissected from *spe-15(hc75);him-5(e1490)* males expressing NMY-2::GFP at 20°C. White arrows point to NMY-2 puncta that appeared in spermatids when the RB was formed. The percentage of secondary spermatocytes with the representative pattern is shown at the top right corner. “*n*” indicates the number of secondary spermatocytes that were followed and quantified. Scale bars: 5 μm. DRAQ5, deep red anthraquinone 5; GFP, green fluorescent protein; NMY-2, non-muscle myosin 2; RB, residual body; SPE-15, defective spermatogenesis 15.

In previously reported in vitro culture conditions, primary spermatocytes develop to form spermatids, but most spermatids remain attached to the residual body (RB), which prevents further characterization of spermatid detachment [1]. Here, we optimized the culture medium so that it can support development of spermatocytes until spermatid release (Materials and methods: “In vitro culture of *C*. *elegans* spermatocytes and live-cell imaging”). The control strain in this study was *him-5*, because *him-5* mutant hermaphrodites produce a much higher percentage of male progeny than the wild-type N2 strain [29]. We cultured primary or secondary spermatocytes dissected from *him-5* males in the optimized culture medium and followed spermatid differentiation by time-lapse recordings (Fig 1A). Consistent with a previous report [1], we observed that 1 primary spermatocyte divided into 2 connected secondary spermatocytes in meiosis I, and each secondary spermatocyte initiated meiosis II to produce 2 connected spermatids that undergo differentiation, leading to generation of 2 individual spermatids and 1 residual body (RB) (Fig 1B, dashed boxes, C, S1A Fig and S1 Video). RBs derived from 2 connected secondary spermatocytes fused spontaneously, forming a large RB (Fig 1B). When cultured in the optimized medium at 20°C, all spermatocytes followed by time-lapse recording developed to produce released spermatids, while on average, 3 out of the 4 spermatids derived from each primary spermatocyte separated from the RB in vitro (Fig 1B). When cultured at 25°C, spermatocytes appeared to develop normally, but spermatid release was reduced significantly, which suggests that elevated temperature affects spermatid release in vitro (Figs 2B and S2D).

By time-lapse recording, we found that a cleavage furrow was formed at the midpoint of the 2 haploid nuclei in meiosis II (Fig 1B and 1C, white arrows). Instead of further contracting, the furrow extended towards the 2 spermatid poles (Fig 1B, double-headed arrow), leading to gradual expansion of the RB (Fig 1B and S1 Video). Later on, new cleavage furrows formed between the spermatids and the RB, which contracted continuously to separate the individual spermatids from the RB (Fig 1B, yellow arrow, S1 Video). Because the first cleavage furrow did not result in membrane fission, it is referred to as the pseudo-cleavage furrow from now on and is thus distinguished from the cleavage furrow formed at the final cytokinesis step.

The differential partitioning of cellular components during spermatid differentiation has been observed previously by electron microscopy and immunohistochemistry [1, 3, 4, 16]. However, it is unclear whether and how the cytoplasm segregation coordinates with RB formation. By live-cell labeling and time-lapse recording, we observed that chromosomes labeled by green fluorescent protein (GFP)-tagged histone 2B (H2B) divided normally in meiosis and were efficiently segregated into differentiating spermatids in vitro (Fig 1C). Mitochondria were first partitioned into 2 domains separated by the pseudo-cleavage furrow and were excluded from RBs during RB expansion (Fig 1D and S2 Video). Ribosomes labeled by ribosomal protein 5, large subunit (RPL-5)::GFP were observed in the cytoplasm and at the cortex of spermatocytes (Fig 1E). RPL-5::GFP became enriched at the pseudo-furrow site and was fully segregated into the expanding RB (Fig 1E and S3 Video). We followed cytoskeleton dynamics in this process by silicon-rhodamine (SiR)-actin or SiR-tubulin staining, which had no notable effect on the progression of RB formation and spermatid release in vitro (S1B Fig). F-actin labeled by SiR-actin or Lifeact was observed throughout the entire cortex of the dividing secondary spermatocytes. Actin filaments became enriched at the pseudo-furrow site and the cortex of the RB during furrow ingression and RB expansion, which appeared to involve continuous centripetal movement of F-actin from the spermatid poles (Fig 1F, white line and white arrow, S1C Fig and S4 Video). Sealing of the cortical actin in the RB occurred when all the actin had moved into the RB (Fig 1F, yellow arrow, S1C Fig), which explains the extremely low levels of actin in released spermatids. We labeled microtubules by SiR-tubulin or GFP fusion of tubulin beta 2 (GFP::TBB-2) and found that increasing amounts of microtubules emanated from the gamma tubulin (TBG-1)-positive microtubule organizing center (MTOC) while the signal from the spindle gradually diminished (S1D and S1E Fig). The microtubule bundles were subsequently released from the MTOC and transported into the RB (S1D and S1E Fig), consistent with previous observations in fixed and live spermatocytes [30, 31]. Most TBG-1 was also transported into RBs, leaving only weak TBG-1 spots in spermatids (S1E Fig, yellow arrow). Altogether, our live-cell imaging of in vitro cultured spermatocytes reveals previously unobserved dynamic processes during spermatid differentiation, including expansion of the pseudo-cleavage furrow, segregation of mitochondria and ribosomes during RB expansion, dynamics of cytoskeleton components, and cytokinesis that separates spermatids from the RB.

### Myosin II and myosin VI play distinct roles to regulate RB formation

Our data suggest that the pseudo-cleavage furrow expands to promote RB formation (Fig 1B and S1 Video). We examined the dynamics of non-muscle myosin 2 (NMY-2), the heavy chain of non-muscle myosin II, which is required for cleavage furrow formation in cytokinesis. After the onset of meiosis II, NMY-2 puncta were observed throughout the entire cell cortex but were concentrated at the spot where 2 secondary spermatocytes were connected (Fig 2A). NMY-2 was then enriched at the site where the pseudo-cleavage furrow formed (Fig 2A, white arrow). The cortical NMY-2 at the pseudo-cleavage furrow extended towards the 2 spermatid poles (Fig 2A, double-headed arrow), concomitant with RB expansion (Fig 2A and S5 Video). *nmy-2(ne3409)* is a temperature-sensitive mutant allele of *nmy-2*, which results in strong loss-of-function (lf) phenotypes in embryogenesis [32]. We found that pseudo-cleavage furrow formation was abolished in *nmy-2(ne3409)* spermatocytes at the nonpermissive temperature of 25°C, indicating that NMY-2 function is required for formation of the pseudo-cleavage furrow (Fig 2B, arrows, S6 Video).

We next examined the role of actin in this process as both NMY-2 and F-actin accumulated at the pseudo-furrow site and the cortex of the expanding RB (S2A Fig), and cytochalasin B is reported to inhibit cell division during spermatogenesis [4]. Latrunculin A disrupts actin filaments by inhibiting actin polymerization. We found that Latrunculin A treatment of secondary spermatocytes prior to appearance of the pseudo-cleavage furrow prevented furrow formation (Fig 2C, white arrows), while treatment of secondary spermatocytes that already had a pseudo-cleavage furrow led to suppression of furrow expansion (Fig 2D, yellow arrows). Thus, actin filaments are required for pseudo-cleavage furrow ingression and subsequent expansion. The actin filaments and myosin II may form actomyosin to power the ingression and expansion processes.

In *him-5* males, RBs were formed through active expansion of the pseudo-cleavage furrow towards the spermatid poles (Fig 2A and 2B). In *nmy-2* mutants, however, pseudo-cleavage furrows did not form, but spermatids budded gradually from the cell body, leaving a structure morphologically similar to the RB (Fig 2B, lines, S6 Video). Thus, myosin II-independent mechanisms may mediate spermatid budding in *nmy-2(ne3409)*. To further test this, we examined whether SPE-15, the *C*. *elegans* myosin VI, is involved in this process. SPE-15 is highly expressed during spermatogenesis and has been implicated in cytoplasm segregation [16, 33]. We constructed double mutants that lack functions of both NMY-2 and SPE-15 and found that spermatid budding was completely suppressed (Figs 2E and S2B). This suggests that SPE-15 function is important for spermatid budding in *nmy-2* mutants. In *spe-15(hc75) nmy-2(ne3409)* double mutants, meiosis II was initiated, and chromosomes were segregated, but formation of spermatids and the RB was completely blocked (Fig 2E and S7 Video). We next generated endogenously expressed GFP::SPE-15 by clustered regularly interspaced short palindromic repeats/CRISPR-associated protein 9 nuclease (CRISPR/Cas9). GFP::SPE-15 accumulated mainly in the nucleus of primary spermatocytes, and was released later into the cytosol, probably due to nuclear envelope breakdown (S2C Fig). After the onset of meiosis II, GFP::SPE-15 was recruited to the cell cortex and moved from the spermatid pole to the spermatid–RB boundary during RB expansion (Fig 2F and S8 Video). In *nmy-2(ne3409)*, SPE-15 movement to the spermatid–RB boundary was unaffected, and it occurred concomitantly with spermatid budding (Fig 2G and S9 video). These data suggest that directional movement of myosin VI may promote spermatid budding in *nmy-2* mutants. Similarly, loss of SPE-15 did not affect enrichment of NMY-2 at the pseudo-furrow site, its expansion towards the spermatid poles, or the accompanying RB expansion (Fig 2H and S10 Video). We found that NMY-2 was completely restricted to the RB in *him-5* worms but diffused into spermatids in *spe-15* mutants, which indicates that SPE-15 is required to restrict NMY-2 within RBs (Fig 2H). Collectively, these data indicate that myosin II and myosin VI regulate spermatid and residual body formation by mediating RB expansion and spermatid budding, respectively.

### Myosin II and myosin VI regulate partitioning of cellular components in different manners

**Fig 3:**
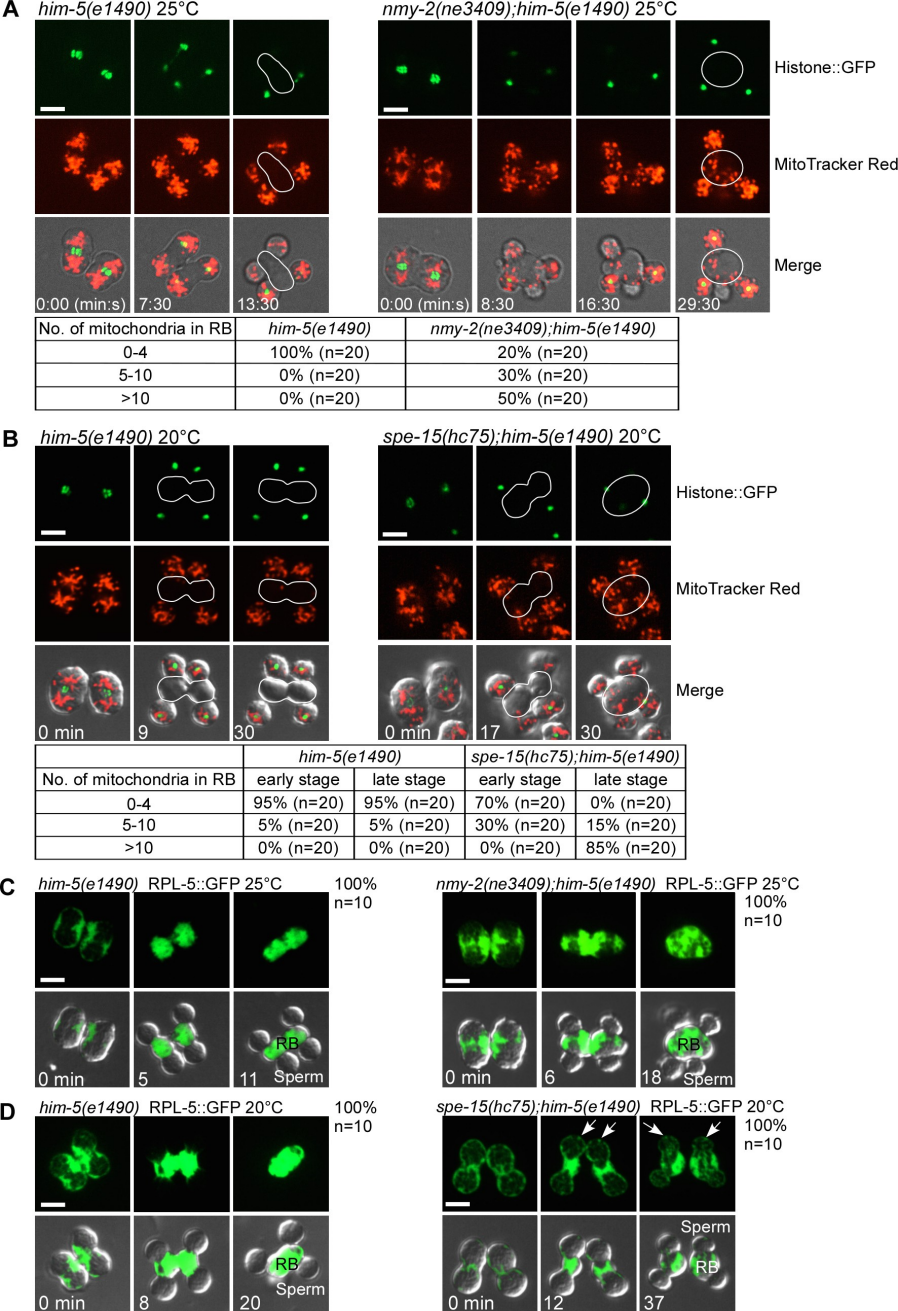
Myosin II and myosin VI regulate cytoplasm segregation in different manners. (A) Time-lapse analysis of meiosis and differentiation in 2 connected secondary spermatocytes dissected from *him-5(e1490)* and *nmy-2(ne3409);him-5(e1490)* males expressing Histone::GFP and stained by MitoTracker Red at the nonpermissive temperature (25°C). Numbers of mitochondria in RBs were quantified, and the results are shown in the table below the images. Underlying data can be found in S1 Data. (B) Time-lapse images of meiosis and differentiation in 2 connected secondary spermatocytes dissected from *him-5(e1490)* and *spe-15(hc75);him-5(e1490)* males expressing Histone::GFP and stained by MitoTracker Red at 20°C. Numbers of mitochondria in newly formed RBs and late RBs were quantified, and the results are shown in the table below the images. Underlying data can be found in S1 Data. (C and D) Time-lapse images of meiosis and differentiation in 2 connected secondary spermatocytes dissected from the indicated strains expressing RPL-5::GFP at 25°C (C) or 20°C (D). Arrows point to cortical ribosomes retained in spermatids of *spe-15(hc75);him-5(e1490)* animals. The percentage of secondary spermatocytes with the representative pattern is shown at the top right corner. *n* indicates the number of secondary spermatocytes that were followed. Scale bars: 5 μm. GFP, green fluorescent protein; RB, residual body; RPL-5, ribosomal protein 5, large subunit.

We found that differential partitioning of cellular components occurs during residual body formation, leading to segregation of mitochondria into spermatids and ribosomes into RBs (Fig 1D and 1E and S2 and S3 Videos). In *him-5* worms, mitochondria were excluded from RBs during NMY-2/RB expansion (S3A Fig). In *nmy-2(ne3409)*, RB expansion was disrupted, and significantly more mitochondria were retained in RB-like structures than in *him-5* (Fig 3A and S11 Video). Mitochondria in *spe-15(hc75)* were segregated normally into spermatids during RB expansion but returned to RBs at a later stage (Fig 3B). Thus, myosin II but not myosin VI is required for the initial segregation of mitochondria into spermatids. RPL-5::GFP was quickly sorted into RBs in *him-5*, and the sorting was not impaired during spermatid budding in *nmy-2(ne3409)* (Fig 3C). In *spe-15* mutants, however, a significant proportion of RPL-5::GFP was retained in the cortex and cytosol of spermatids during residual body formation, indicating that ribosome segregation into RBs is impaired (Figs 3D and S3B and S12 Video). The cytoskeleton components actin and tubulin were moved to and enriched in RBs in *him-5* worms. However, actin and tubulin were present in spermatids in *spe-15* mutants (S3C Fig). This is consistent with the previous report [16] and suggests that segregation of actin and tubulin into RBs is affected in *spe-15(lf)*. In line with this, transport of microtubules to RBs was impaired in *spe-15*, but it was not obviously affected in *nmy-2(ne3409)* mutants (S3D and S3E Fig). Collectively, these data indicate that myosin II and myosin VI have distinct effects on cytoplasm segregation, which is in good agreement with the directional movement of the 2 myosins during spermatid budding and RB expansion. Myosin II extends towards the 2 spermatid poles and is required for segregation of mitochondria into spermatids, while myosin VI moves from the spermatid poles to the RB and is involved in transporting ribosomes and cytoskeleton components into the RB.

### Myosin VI but not myosin II is important for cytokinesis that separates spermatids from the RB

**Fig 4:**
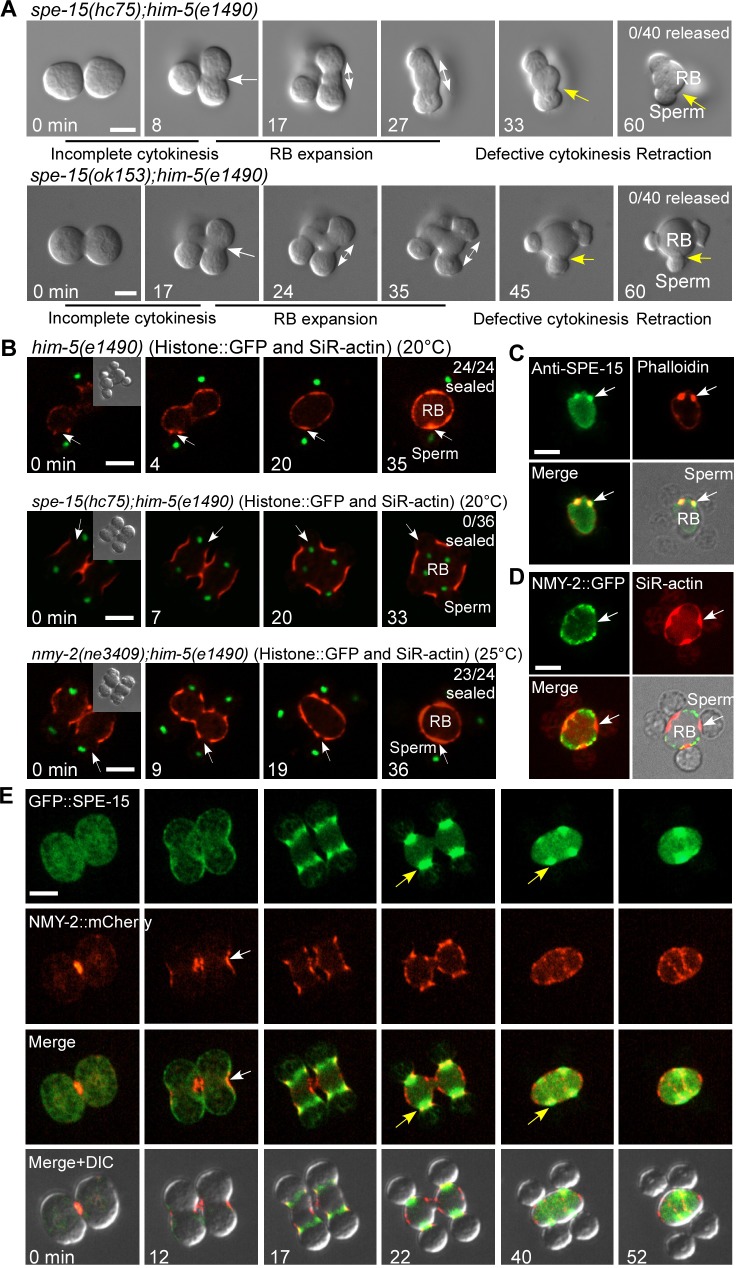
Spermatid release is achieved through myosin VI–mediated cytokinesis. (A) Time-lapse analysis of meiosis and differentiation in 2 connected secondary spermatocytes dissected from *spe-15(hc75);him-5(e1490)* and *spe-15(ok153);him-5(e1490)* males. White arrows indicate the pseudo-cleavage furrow between 2 undifferentiated spermatids. Double-headed arrows indicate RB expansion during spermatid differentiation. Yellow arrows point to the spermatid–RB boundary and indicate defective cytokinesis. Numbers of released spermatids/total spermatids were quantified and are shown in the top right corner. (B) Time-lapse analysis of spermatids and RBs that undergo cytokinesis in the indicated strains expressing Histone::GFP and stained by SiR-actin. White arrows indicate sites of cytokinesis. Numbers of sealed spermatids/total spermatids were quantified and are shown in the top right corner. DIC images of spermatids and RBs at the start time point (0 min, insets) are shown. (C and D) Light and fluorescence images of an RB with 4 attached spermatids stained by anti-SPE-15 antibody and phalloidin (C) or expressing NMY-2::GFP and stained by SiR-actin (D). Arrows point to the connection between the spermatid and RB. (E) Time-lapse images of meiosis and differentiation in 2 connected secondary spermatocytes dissected from *him-5* males expressing GFP::SPE-15 and NMY-2::mCherry. White arrows indicate the pseudo-cleavage furrow with enriched NMY-2. Yellow arrows indicate accumulation of SPE-15 at the cleavage furrow between spermatids and RB. Scale bars: 5 μm. DIC, differential interference contrast; GFP, green fluorescent protein; NMY-2, non-muscle myosin 2; RB, residual body; SiR, silicon-rhodamine; SPE-15, defective spermatogenesis 15.

In *nmy-2* mutants, spermatids budded and eventually separated from the RB-like structure as in *him-5*, suggesting that the final cytokinesis may occur independent of NYM-2 (Figs 2B and S2D). We examined spermatid differentiation in *spe-15* mutants. In *spe-15(lf)*, both pseudo-cleavage furrow formation and RB expansion were unaffected (Fig 4A, white arrows), but membrane constriction between spermatids and RBs was not completed (Fig 4A, yellow arrows, S13 Video). We further examined membrane constriction as indicated by the closure of cortical actin visualized through SiR-actin staining (Fig 1F). In *him-5* worms, all spermatid–RB connections (24 of 24) were sealed quickly, whereas none of them (0 of 36) was sealed in *spe-15* mutants (Fig 4B). The closure of cortical actin was unaffected in *nmy-2(ne3409)* (23 of 24), suggesting that membrane constriction between spermatids and RBs was normal (Fig 4B). Similar results were observed when cortical actin was detected by Lifeact (S4 Fig). Consistent with this, SPE-15, but not NMY-2, was enriched on the actin patch at the cleavage site (Fig 4C and 4D).

By monitoring the dynamics of GFP::SPE-15, we found that SPE-15 migrated from the spermatid poles to the RB during spermatid budding and was enriched at the site where membrane constriction occurred (Fig 2F, yellow arrows, S8 Video). This temporal localization pattern is consistent with the role of SPE-15 in regulating membrane constriction and spermatid release. We next compared the dynamics of myosin II and myosin VI in worms that carry endogenously expressed NMY-2::mCherry and GFP::SPE-15. NMY-2, but not SPE-15, was enriched at the pseudo-cleavage furrow between 2 haploid spermatids (Fig 4E, white arrows, S14 Video). When the RB was formed, NMY-2 appeared as punctate structures distributed throughout the entire cortex of the RB, whereas SPE-15 was specifically enriched at the constriction and cleavage site at which spermatids and the RB are separated (Fig 4E, yellow arrows, S14 Video). Altogether, these data indicate that myosin VI, but not myosin II, is required for the final cytokinesis.

Defective cytokinesis may lead to retraction of polarized spermatids and generation of nucleus-containing residual bodies. Nucleus-containing RBs may be recognized and removed by the phagocytic pathway, similar to nucleus-free RBs (S5A Fig) [7]. We examined accumulation of nucleus-containing RBs in cell death abnormality 1 (*ced-1*) mutants, which lack the phagocytic receptor CED-1 and are defective in RB clearance [7]. We found that very few residual bodies contained nuclei in *ced-1;him-5* (6%), indicating normal cytokinesis and spermatid release (S5B Fig). By contrast, all RBs in *ced-1spe- 15(hc75)* contained nuclei, indicating that loss of SPE-15 causes defective cytokinesis in vivo (S5B Fig). Loss of *spe-15* also affected spermatid activation in vitro, likely due to incorrect cytoplasm segregation (S5C Fig) [16]. *spe-15(lf)* hermaphrodites produced much fewer self-progeny than wild-type worms (S5D Fig). After mating with *him-5* males, the number of cross progenies derived from wild-type and mutant worms was similar, suggesting that the infertility is caused by defects in spermatids but not oocytes (S5E Fig). In line with this, *spe-15(lf)* hermaphrodites produced a high percentage of unfertilized eggs, confirming spermatid defects (S5F Fig). These data indicate that myosin VI–mediated cytokinesis is essential for spermatid release and fertility.

### Myosin VI and F-actin form an actomyosin VI contractile ring to promote cytokinesis

**Fig 5:**
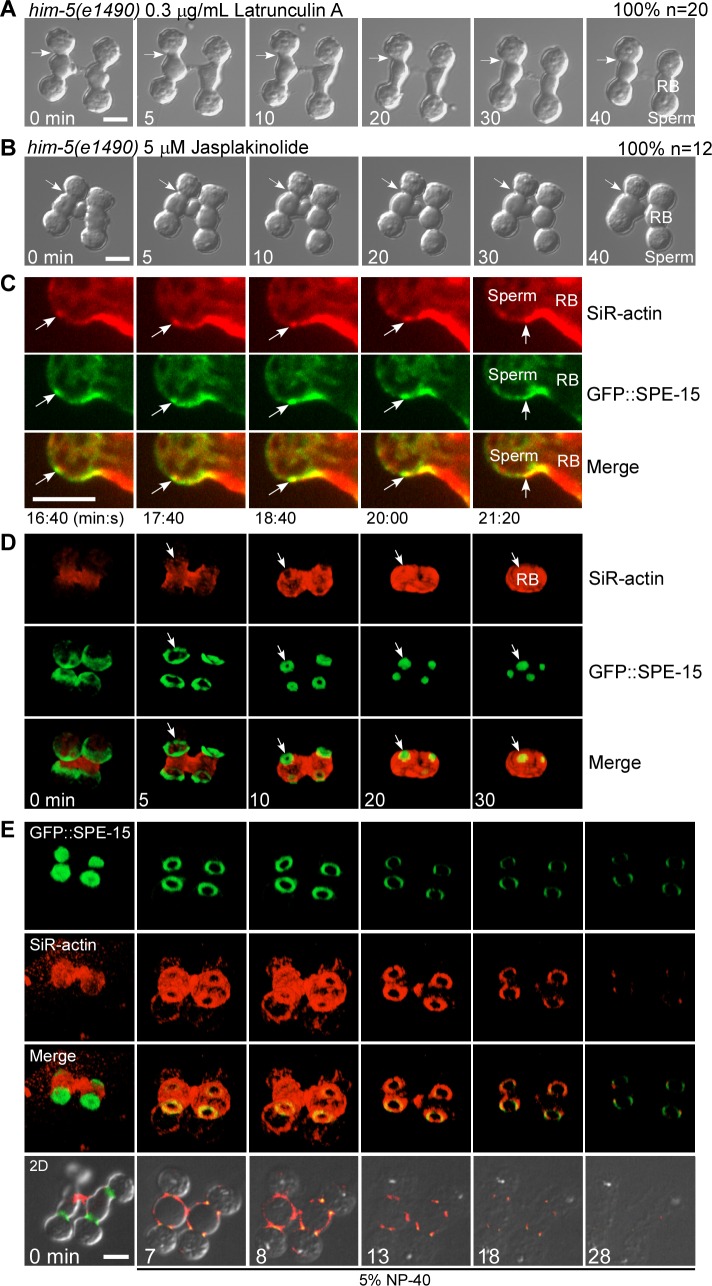
Myosin VI and F-actin form an actomyosin VI contractile ring to control cytokinesis. (A and B) Time-lapse images of the secondary spermatocytes (dissected from *him-5* males) that undergo cytokinesis in the presence of Latrunculin A or Jasplakinolide. Arrows indicate the spermatid–RB connection and defective cytokinesis. The percentage of connections with the representative pattern is shown at the top right corner. *n* indicates the number of connections that were followed and quantified. (C) Time-lapse images showing differentiation of a spermatid dissected from a *him-5* male expressing GFP::SPE-15 and further stained by SiR-actin. White arrows indicate movement of a SPE-15–positive SiR-actin punctum from the spermatid pole to the RB. (D) Reconstituted 3D time-lapse images showing differentiation of connected spermatids dissected from *him-5* males expressing GFP::SPE-15 and further stained by SiR-actin. Arrows indicate contraction of the SPE-15 ring and sealing of the cortical actin. (E) Reconstituted 3D time-lapse images showing resistance of the actomyosin VI ring to detergent treatment; 2D images in the bottom row show disruption of spermatids and RBs by detergent. Scale bars: 5 μm. GFP, green fluorescent protein; RB, residual body; SiR, silicon-rhodamine; SPE-15, defective spermatogenesis 15.

We found that both Latrunculin A and Jasplakinolide treatment suppressed membrane constriction as in *spe-15* mutants, which indicates that membrane constriction requires polymerization and depolymerization of actin filaments (Fig 5A and 5B). We examined the dynamics of GFP::SPE-15 and F-actin simultaneously and found that SPE-15 was recruited to the cell cortex upon entry into meiosis II, concomitant with the temporal increase of cortical actin (S6A Fig). Then, SPE-15 and F-actin comigrated from the spermatid pole to the RB and accumulated at the site where membrane constriction occurred (Figs 5C and S6B and S6C). Thus, SPE-15 and F-actin may act together to regulate cytokinesis between spermatids and the RB. By visualizing SPE-15 and F-actin in 3 dimensions, we observed that SPE-15 formed ring-like structures, and contraction of the ring was concomitant with sealing of the cortical actin (Fig 5D). We treated spermatocytes that contain enriched SPE-15 at the spermatid–RB boundary with 5% NP40. Both spermatids and RBs were permeabilized by NP40, whereas the SPE-15/F-actin rings remained (Fig 5E and S15 Video). We found that the subcortical actin cytoskeleton in RBs disappeared much faster than the SPE-15/F-actin ring during treatment (Fig 5E and S15 Video). This suggests that SPE-15 and the associated actin filaments form stable actomyosin VI rings, which may contract to promote cytokinesis.

### GIPC cooperates with myosin VI to regulate actomyosin VI ring formation and spermatid release

**Fig 6:**
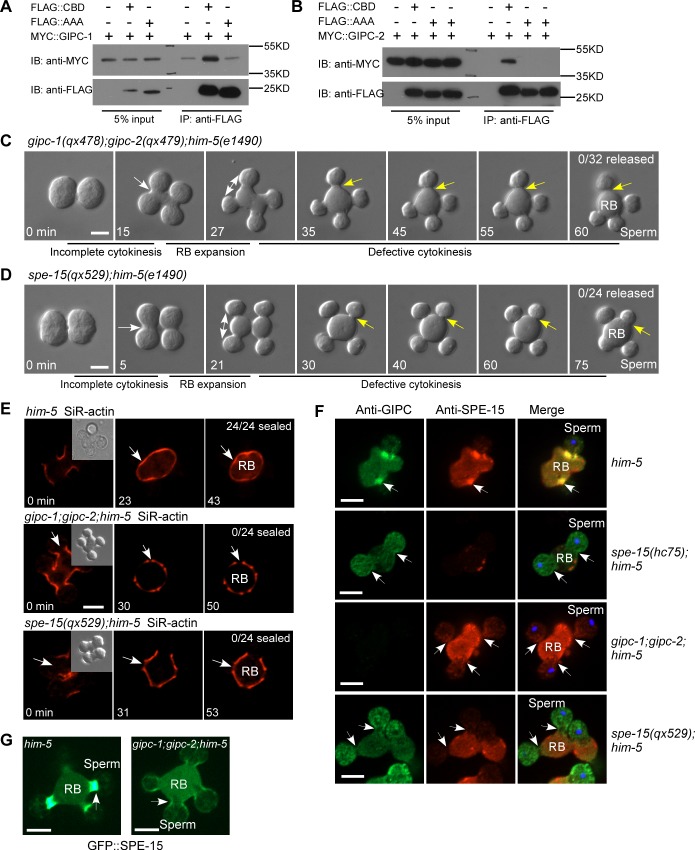
GIPC interacts with myosin VI to regulate actomyosin VI ring formation and spermatid release. (A and B) GIPC-1 (A) or GIPC-2 (B) is coprecipitated by the CBD of wild-type SPE-15 but not the AAA SPE-15 mutant (R1067A, R1068A, and L1069A). (C and D) Time-lapse images of meiosis and differentiation in 2 connected secondary spermatocytes dissected from *gipc-1;gipc-2;him-5* (C) or *spe-15(qx529);him-5(e1490)* (D) males. White arrows indicate the pseudo-cleavage furrow between 2 undifferentiated spermatids. Double-headed arrows indicate RB expansion during spermatid differentiation. Yellow arrows point to the spermatid–RB connections and indicate cytokinesis defects. Numbers of released spermatids/total spermatids were quantified and are shown in the top right corner. (E) Time-lapse analysis of the cytokinesis process in SiR-actin–stained spermatids/RBs from the indicated strains. White arrows indicate sites of cytokinesis. Numbers of sealed spermatids/total spermatids were quantified and are shown in the top right corner. DIC images of spermatids and RBs at the start time point (0 min, inset) are shown. (F) Fluorescence images of RBs with attached spermatids in the indicated strains stained by anti-GIPC and anti-SPE-15 antibody. White arrows point to spermatid–RB connections. (G) Fluorescence images of an RB with 4 attached spermatids dissected from *him-5* or *gipc-1;gipc-2;him-5* males expressing GFP::SPE-15. White arrows point to a spermatid–RB connection. Scale bars: 5 μm. CBD, cargo-binding domain; DIC, differential interference contrast; GFP, green fluorescent protein; GIPC, RGS-GAIP-interacting protein C terminus; IB, immunoblotting; IP, immunoprecipitation; RB, residual body; SiR, silicon-rhodamine; SPE-15, defective spermatogenesis 15.

Myosin VI regulates various membrane trafficking processes through interactions with different partners. We performed yeast two-hybrid screening to search for SPE-15–interacting proteins and identified GIPC-1 and GIPC-2. They are members of the RGS-GAIP-interacting protein C terminus (GIPC) family, which act as cargo adaptors to load a variety of cargos for myosin VI–dependent endosomal trafficking [34]. GIPC-1 and GIPC-2 are almost identical in amino acid sequence and are highly expressed during spermatogenesis (S6D Fig) [33]. We found that both GIPC-1 and GIPC-2 interacted with SPE-15 through its RRL motif, which is located in the cargo-binding domain (CBD) and is known to mediate the interaction of myosin VI with adaptor proteins (Figs 6A and 6B and S6E) [35, 36]. We used CRISPR/Cas9 to generate premature stop-codon mutations of *gipc-1* and *gipc-2* and a point mutation of *spe-15*, *qx529*, in which the RRL motif is mutated to AAA (S6D and S6E Fig). We found that cytokinesis was impeded in *gipc-1;gipc-2* double mutants and in *spe-15(qx529)*. In these worms, the spermatids failed to separate from RBs, and the cortical actin was not sealed (Fig 6C–6E). This suggests that GIPCs and their interactions with SPE-15/myosin VI are important for myosin VI–mediated cytokinesis for spermatid release.

We generated an antibody that recognizes both GIPC-1 and GIPC-2 and stained GIPC and SPE-15 simultaneously. GIPC and SPE-15 both accumulated at the constriction region between spermatids and the RB, while only faint signals were detected at the spermatid poles (Figs 6F and S6F). Loss of GIPCs disrupted accumulation of SPE-15 between spermatids and the RB, indicating that GIPC is required for formation of the actomyosin VI contractile ring (Fig 6F and 6G). Enrichment of GIPC at the cleavage region was completely abolished in *spe-15(hc75)* mutants, indicating that GIPC localization requires SPE-15/myosin VI (Fig 6F). In agreement with this, neither SPE-15 nor GIPC was enriched at the cleavage region in *spe-15(qx529)* mutants, in which the SPE-15-GIPC interaction is disrupted (Fig 6F). Altogether, these data indicate that GIPC and SPE-15 cooperate to regulate contractile ring formation and spermatid release.

We found that cytoplasm segregation was impaired in *gipc-1;gipc-2* double mutants and *spe-15(qx529)* mutants (S3B, S3C and S3F Fig). Moreover, *gipc-1;gipc-2* and *spe-15(qx529)* contained a high percentage of nucleus-containing RBs, consistent with defective cytokinesis in vivo (S5B Fig). Furthermore, *gipc-1;gipc-2* and *spe-15(qx529)* affected spermatid activation in vitro and caused infertility due to defects in spermatids (S5C–S5F Fig). These phenotypes resembled those in *spe-15(lf)*, which further suggests that GIPCs act as the key regulator of SPE-15/myosin VI in spermatid differentiation.

## Discussion

**Fig 7:**
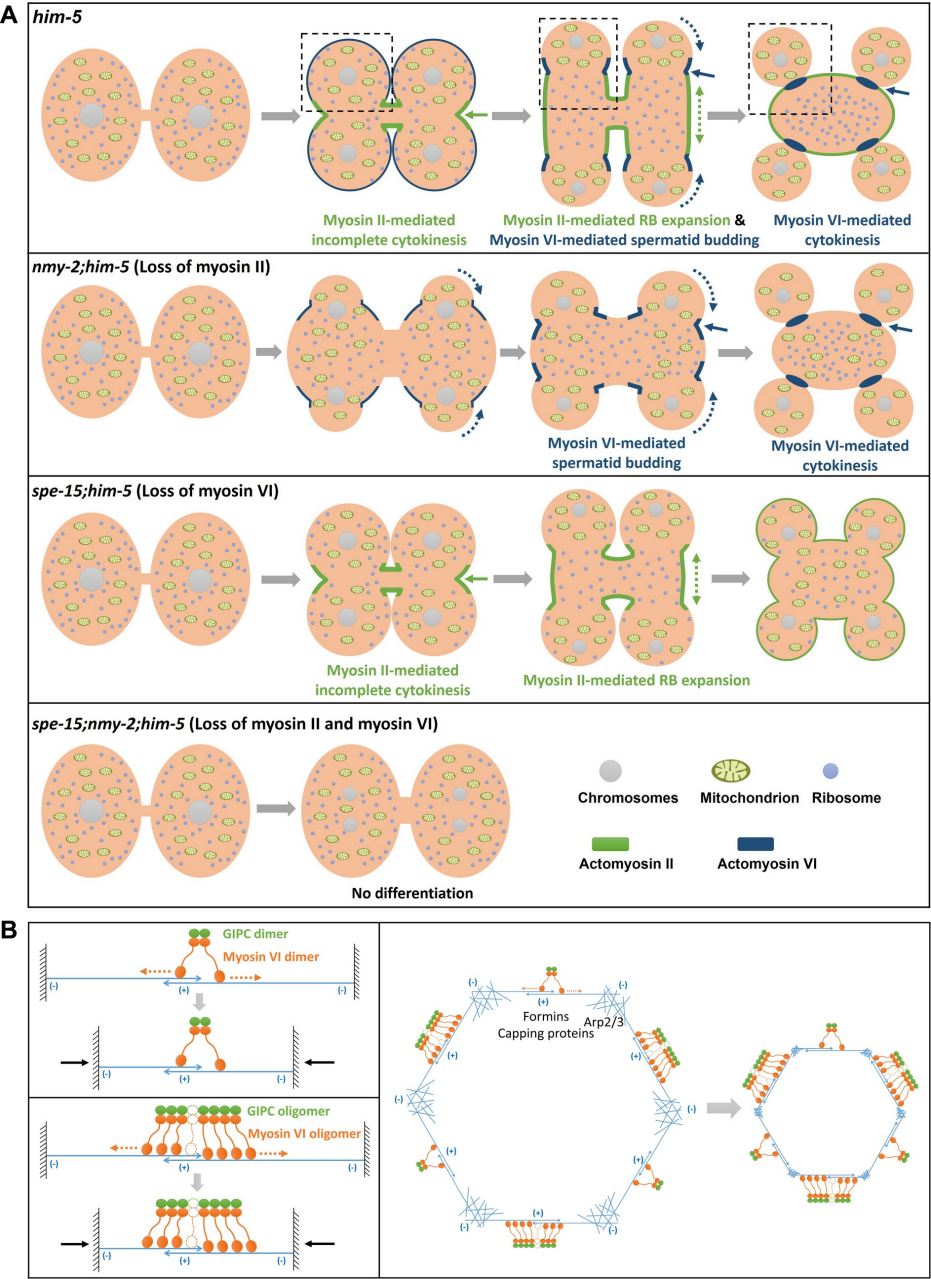
Proposed models showing the role of myosin II and myosin VI in spermatid differentiation. (A) Proposed model of the role of myosin II and myosin VI in spermatid differentiation. Myosin II and myosin VI play distinct roles to regulate RB formation and cytoplasm segregation. Dashed boxes show the differentiation process of a single haploid spermatid in *him-5* animals. Residual body formation is achieved by myosin II–mediated RB expansion and myosin VI–mediated spermatid budding. Spermatids are separated from RBs by myosin VI–mediated cytokinesis. Cellular contents are differentially segregated into spermatids and RBs during RB expansion, regulated by myosin II and myosin VI, respectively. Green arrows point to the pseudo-cleavage furrow that is enriched in actomyosin II. Blue arrows indicate accumulation of actomyosin VI at the cleavage furrow between the spermatid and the RB. Green dashed arrows and blue dashed arrows indicate the movements of NMY-2 and SPE-15, which are in opposite directions. In *spe-15 nmy-2* double mutants, spermatid differentiation is completely blocked. (B) Proposed model of actomyosin VI ring formation and contraction in separating spermatids from the RB. GIPC may promote myosin VI dimerization or oligomerization, which is required for actomyosin VI ring formation. Head domains in SPE-15 dimers or oligomers may localize to anti-parallel actin bundles. Their movement towards the minus end of actin bundles may generate a pulling force and induce actin sliding, which drive contraction of the actomyosin VI ring. Other actin regulators, including formins, Arp2/3, and capping proteins, may regulate the architecture and dynamics of the actin network that are required for actomyosin VI ring formation and contraction. Arp2/3, actin-related proteins 2 and 3; GIPC, RGS-GAIP-interacting protein C terminus; NMY-2, non-muscle myosin 2; RB, residual body; SPE-15, defective spermatogenesis 15.

Using an approach combining live-cell imaging and genetic manipulation in an optimized in vitro culture system, we monitored and investigated spermatid differentiation with high temporal and spatial resolution, which reveals previously unseen cell biological processes that drive RB expansion, spermatid budding, cytoplasm segregation, and cytokinesis (Figs 1A and 7A). Our data demonstrate that myosin II and myosin VI regulate residual body formation by mediating RB expansion and spermatid budding, respectively (Fig 7A). In the meantime, cellular contents are differentially segregated into spermatids and RBs, which requires functions of myosin II and myosin VI, respectively (Fig 7A). Finally, spermatids are separated from RBs by myosin VI–mediated cytokinesis (Fig 7A and 7B).

Our data suggest that movement of the 2 myosins in opposite directions may drive RB expansion and spermatid budding and, at the same time, promote partitioning of cellular components into RBs and spermatids (Fig 7A). It is possible that pseudo-cleavage furrow extension towards the spermatid poles, powered by NMY-2/myosin II and F-actin, excludes organelles like mitochondria from the central region during RB formation and thus promotes mitochondrial segregation into spermatids (S3A Fig). We suspect that mitochondria may connect with astral microtubules that associate with the cell cortex. The astral microtubules and the associated mitochondria may be excluded from the central region during RB/NMY-2 expansion (S3A and S3D Fig, 6 min), which ensures that mitochondria are not partitioned into RBs. In *spe-15(lf)*, the initial segregation of mitochondria into spermatids is not obviously affected, indicating that SPE-15 is not required for active positioning of mitochondria (Fig 3B). At the late stage, however, spermatids in *spe-15(lf)* are not separated from the RB, and mitochondria-containing spermatids retract, causing appearance of mitochondria in the RB (Fig 3B). This suggests that timely myosin VI–mediated cytokinesis is essential for maintaining chromosomes and mitochondria in spermatids. On the other hand, movement of SPE-15/myosin VI with the cortical actin from spermatids to RBs may promote spermatid budding and transport of cytoskeleton components and ribosomes into RBs (Figs 5C and 7A). Moreover, enrichment of SPE-15 and F-actin at the RB–spermatid boundary may serve as a barrier to prevent entry of cortical actin and NMY-2 into spermatids. Future studies should address whether and how mitochondria may connect with astral microtubules and whether SPE-15 achieves its segregation function by mediating cross-linking of ribosomes and microtubules with actin filaments.

Our data show that spermatid differentiation shares common properties with normal cytokinesis but has unique features. As in cytokinesis, myosin II and F-actin form an actomyosin II ring to drive cleavage furrow ingression at the equatorial region of the dividing secondary spermatocyte. However, instead of further constricting membranes at the furrow site as in cytokinesis, the actomyosin II ring expands towards the 2 spermatid poles, which may provide the force for RB expansion (Figs 2A and 7A). The positioning of the actomyosin II contractile ring and its subsequent extension may be directed by the anaphase spindle, but how this process is regulated remains unclear.

We found that the final release of spermatids is independent of myosin II but is carried out by myosin VI–mediated cytokinesis (Fig 7). In this process, SPE-15/myosin VI and F-actin form detergent-resistant actomyosin VI rings, and contraction of the actomyosin VI ring may drive membrane constriction for spermatid release (Fig 5C–5E and 7B). The upstream signal(s) that trigger(s) SPE-15 movement towards RBs and contraction of the actomyosin VI ring is unclear and requires further investigations.

Myosin VI is capable of inducing actin network deformation in vitro [37]. Here, we found that actomyosin VI contractile rings can be formed in vivo and are responsible for cytokinesis and spermatid release. It is reported that antiparallel actin bundles and branched meshwork, but not parallel bundles, are selectively contracted by myosin VI in vitro [37, 38]. In this process, reconstituted double-headed myosin VI is used, which triggers continuous contraction by tethering and sliding neighboring antiparallel filaments [38]. Here, we found that SPE-15/myosin VI associates with the cargo adaptor GIPC, and the interaction is required for actomyosin VI ring formation and spermatid release. Myosin VI is known to undergo GIPC-dependent dimerization or oligomerization in endocytosis and endocytic transport [25, 39–42]. Therefore, GIPC may promote SPE-15/myosin VI dimerization to facilitate actomyosin VI ring formation and contraction like reconstituted double-headed myosin VI in vitro (Fig 7B) [38]. In addition, SPE-15 oligomers may act as myosin II minifilaments to tether and slide antiparallel actin bundles, thus generating the force for contraction (Fig 7B). We found that both Latrunculin A and Jasplakinolide treatment suppressed membrane constriction between spermatids and RBs (Fig 5A and 5B). This suggests that actin polymerization and depolymerization are both important for actomyosin VI ring contraction, similar to actomyosin II rings [43]. Formin, actin-related proteins 2 and 3 (Arp2/3), profilin, and capping proteins may collaborate to control actin network architecture and dynamics in this process (Fig 7B). In-depth analyses are needed to determine which factors are involved and how they act. Future studies should also address whether myosin VI–mediated membrane transport processes may participate in spermatid release as in cytokinesis of mitotic cells [44].

By utilizing two myosins to mediate incomplete and complete cytokinesis in a sequential order, a series of events including meiosis II, RB formation, cytoplasm segregation, and spermatid release are precisely coupled during *C*. *elegans* spermatid differentiation (Fig 7A). Similar mechanisms may be utilized throughout evolution and employed in other processes involving actin-based regulations such as transport of the adaptor protein Miranda during asymmetric cell division of *Drosophila* neuroblasts [45]. Moreover, our finding that myosin VI forms actomyosin VI rings to mediate cytokinesis and spermatid release may provide new insights into other myosin VI–controlled cellular processes [46–48].

## Materials and methods

### *C*. *elegans* strains

Strains of *C*. *elegans* were cultured and maintained using standard protocols. N2 Bristol strain hermaphrodites and *him-5(e1490)* (linkage group [LG] V) males were used as the wild-type strain. The following mutant strains were used in this work: LG I: *spe-15(hc75)*, *spe-15(ok153)*, *spe-15(qx529)*, *nmy-2(ne3409)*, and *ced-1(e1735)*; LG III: *gipc-1(qx478)*; and LG IV: *gipc-2(qx479)*. The reporter strains used in this study include *cp13* (NMY-2::GFP) [49], *qx654* (NMY-2::mCherry), *qx506* (GFP::SPE-15), *qx548*(RPL-5::GFP), *qxIs91* (P_*ced-1*_CED-1::GFP), *zbIs2* (P_*pie-1*_LifeACT::RFP), *stIs10027* (P_*his-72*_HIS-72::GFP), *tjIs54* (P_*pie-1*_GFP::TBB-2 + P_*pie-1*_2xmCherry::TBG-1), and *itIs37*(P_*pie-1*_mCherry::H2B) [50].

*spe-15(qx529)*, *gipc-1(qx478)*, *gipc-2(qx479)*, *qx506*, *qx654*, and *qx548* were generated by genome editing using CRISPR/Cas9 according to the method below. *spe-15(qx529)* contains the amino acid substitutions R1067A, R1068A, and L1069A. *gipc-1(qx478)* and *gipc-2(qx479)* contain premature stop codons after E44 and V53, respectively. *stIs10027* and *itIs37* were kindly provided by Z. Bao (Sloan Kettering Institute, New York, NY). All the other stains were provided by the Caenorhabditis Genetics Center.

*spe-15(hc75)* contains a premature stop codon (W804stop), and *spe-15(ok153)* contains a deletion that results in truncation of the motor domain of the SPE-15a isoform [16]. *nmy-2(ne3409)* is a temperature-sensitive allele that contains the amino acid substitution L981P [32]. The encoded protein is nonfunctional at 25°C. *nmy-2(ne3409)* worms were maintained at 16°C and were switched to 25°C for time-lapse recording and phenotypic analyses.

### Genome editing using CRISPR/Cas9

Genome editing was performed according to the protocol from Paix and colleagues [51]. The 19 bp gene-specific guide sequence was constructed in pDD162 (Addgene #770047)—which has a gRNA backbone and a Cas9 gene—by site-directed mutagenesis using the following primers: PSL129/130 for *gipc-1(qx478)*, PSL139/140 for *gipc-2(qx479)*, PSYC843/844 for *spe-15(qx529)*, PJYH59/60 for *qx506*, PJYH319/320 for *qx654*, and PYBL135/136 for *qx548*. The following repair templates, which are less than 120 nt, were synthesized directly and used as single-stranded templates: PSL131 for *gipc-1(qx478)*, PSL141 for *gipc-2(qx479)*, and PSYC845 for *spe-15(qx529)*. For easier selection of desired mutations, restriction enzyme recognition sites were included in (*Xba* I in *gipc-1*, *Nco* I in *gipc-2*) or excluded from (*EcoR* I in *spe-15*) the repair DNA sequences. Longer double-stranded templates were generated by PCR using primers containing homology arms: PJYH61/62 for *qx506*, PJYH428/429 for *qx654*, and PYBL147/PYBL148 for *qx548*. To prepare the injection mixture, pDD162 containing a gRNA sequence and the Cas9 gene was diluted to a final concentration of 20 ng/μl, short linear DNA templates were diluted to 1 μM, and longer templates were diluted to 50 ng/μl. An oligonucleotide template for *dpy-10* repair and a pDD162 construct containing a *dpy-10* gRNA were included in the injection mixture for F1 screening. F1 worms showing roller or dumpy phonotypes were singled. Worm lysates were used for PCR or PCR followed by restriction enzyme digestion to identify worms containing the desired DNA changes. All strains generated by genome editing were outcrossed 4 times before use. Primers and oligonucleotide templates used for CRIPSPR/Cas9 are included in S1 Table.

### In vitro culture of *C*. *elegans* spermatocytes and live-cell imaging

L4-stage males with rounded tails were picked and placed on fresh OP50-seeded plates and grown for 1 to 2 d. The celibate males were then transferred to a new plate without bacteria and allowed to crawl for a few minutes. Three to 5 μl of Leibovitz's L-15 medium (Gibco 21083–027) containing 15% FBS (Pan p11606) was added onto a clean slide. Three to 5 males were quickly dissected in the medium using 2 needles to release the spermatocytes. A clean coverslip was carefully placed over the sample and then sealed with a mixture of beeswax and Vaseline (1:1) for imaging.

Spermatocytes at different developmental stages were distinguished by differential interference contrast (DIC) morphology observed using a 10× objective. Fluorescence images were captured using a 100× objective (CFI Plan Apochromat Lambda; NA 1.45; Nikon) with immersion oil (type NF) on an inverted fluorescence microscope (Eclipse Ti-E; Nikon) equipped with a spinning-disk confocal scanner unit (UltraView; PerkinElmer) with 488 (emission filter 525 [W50]), 561 (dual-band emission filter 445 [W60] and 615 [W70]), and 635 (dual-band emission filter 485 [W60] and 705 [W90]) lasers. For time-lapse recording, images were captured at intervals ranging from 10 s to 1 min (10 s, 15 s, 20 s, 30 s, or 1 min) at 20°C or 25°C. The room temperature was adjusted to 20°C or 25°C before images were taken. The Z stage was adjusted as required to focus on the middle section of spermatocytes. Images were viewed and analyzed using Volocity software (PerkinElmer). For 3D reconstruction, images in 13 z series (0.5 μm/section) were captured every 1 min at 20°C and were viewed and reconstituted using Volocity software (PerkinElmer).

Time-lapse recording with DIC alone was performed using a 100× objective (Plan-Neofluar; NA 1.40; Carl Zeiss) with Immersol 518F oil (Carl Zeiss) on an Axioimager M2 microscope (Carl Zeiss) equipped with an AxioCam monochrome digital camera (Carl Zeiss). Images were captured every 10 s at 20°C or 25°C. The Z stage was adjusted as required to focus on the middle section of spermatocytes. Images were processed and viewed using ZEN 2 software (Carl Zeiss).

*nmy-2(ne3490)* spermatocytes in the sealed slides were kept at 25°C for at least 2 min before imaging to ensure the protein became nonfunctional. The temperature was maintained at 25°C during time-lapse recording.

### Vital stain labeling and drug treatment

SiR-actin (Spirochrome SC001) and SiR-tubulin (Spirochrome SC002) were used for live-cell imaging with a final concentration of 1 μM. Worms were dissected in culture medium containing 1 μM SiR-actin or SiR-tubulin and incubated for 5 min at 20°C in the dark before imaging in the same medium. Mitochondria in the released spermatocytes were labeled and imaged in culture medium containing 50 nM Mitotracker Red CMXRos (ThermoFisher M7512). Nuclei were labeled with 1 μg/ml Hoechst 33342 (ThermoFisher 62249) or 50 nM DRAQ5 (ThermoFisher 62254) in culture medium.

For drug treatment, a final concentration of 0.3 μg/ml Latrunculin A (Sigma L5163) or 5 μM Jasplakinolide (Abcam ab141409) was utilized. Worms were dissected in culture medium containing Latrunculin A or Jasplakinolide and incubated for 1 to 2 min at 20°C before imaging in the same medium. Temperature was maintained at 20°C during time-lapse recording.

### Detergent treatment

Three to five celibate young males were dissected in 2 μl culture medium containing 2 μM SiR-actin on a glass-bottom dish (NEST 801002). A piece of wet tissue was placed in the dish to prevent medium evaporation. The dish was kept in the dark for 5 min at 20°C before adding 2 μl Matrigel (Corning 356234) to make the medium solid. Images in 13 z series (0.5 μm/section) were captured every 30 s for 30 min at 20°C using a spinning-disk confocal scanner unit (UltraView; PerkinElmer). The amount of 10 μl 5% NP-40 (Fluka 74358) was added on top of the solid medium after the first time point. Images were viewed and analyzed using Volocity software (PerkinElmer).

### Yeast two-hybrid screen

The ProQuest (ThermoFisher) yeast two-hybrid system was used to search for SPE-15 partners using the CBD (Q1031-P1219) of SPE-15 as a bait. SPE-15(CBD)::FLAG was constructed in pDBLeu, and protein expression was determined by western blot. The optimal concentration of 3-amino-1,2,4-triazole was determined by self-activation testing of SPE-15 (CBD). A library of *C*. *elegans* cDNA in pPC86 was then screened using pDBleu-SPE-15(CBD)::FLAG. Transformants were selected on synthetic complete (SC) medium lacking leucine, tryptophan, and histidine (SC-Leu-Trp-His) with optimal 3-amino-1,2,4-triazole for the activation of the reporter gene HIS3. The positive transformations were sequenced to identify the gene.

### Coimmunoprecipitation

FLAG::SPE-15 (CBD), FLAG::SPE-15(CBD) (RRL to AAA), MYC::GIPC-1, and MYC::GIPC-2 were all constructed in pET21b. Proteins were expressed in and purified from *Escherichia coli* BL21 (DE3) using Ni-NTA resin (Qiagen 30210). For coimmunoprecipitation, 4 μg FLAG::SPE-15(CBD) or 8 to 12 μg FLAG::SPE-15(CBD) (RRL to AAA) was mixed with 4 μg MYC::GIPC-1 or MYC::GIPC-2 in 400 μl TBST (50 mM Tris-HCl [pH 7.5], 300 mM NaCl, and 0.1% Tween 20) and rotated at 4°C for 2 h. The amount of 20 μl anti-FLAG M2 magnetic beads (Sigma M8823) was added to each tube and rotated at 4°C for another 4 h. Beads were collected by applying the tube to a magnetic separator. Supernatant was removed carefully. Beads were washed 3 times for 10 min each in TBST before bound proteins were resolved with SDS-PAGE and revealed by western blotting with anti-MYC antibody (rabbit, sc-789; Santa Cruz).

### Antibody generation

Full-length GIPC-2 protein with six histidine residues (plasmid pET21b-MYC::GIPC-2) was expressed in and purified from *E*. *coli* BL21 (DE3) using Ni-NTA resin (Qiagen 30210), then used to raise rabbit polyclonal antibody. The anti-GIPC-2 antibody recognized both GIPC-1 and GIPC-2 in a western blot analysis using recombinant proteins. The antibody was further purified as described previously [52]. A C-terminal fragment of SPE-15a protein (amino acids 838–1219) fused with 6 histidine residues was expressed in and purified from *E*. *coli* BL21 (DE3) using Ni-NTA resin (Qiagen 30210), then used to raise mouse monoclonal antibody.

### Immunostaining

Slides were coated with poly-lysine (5%) and marked with a small circle using a PAP pen after drying. Twenty males were dissected in 20 μl SM buffer (50 mM HEPES, 25 mM KCl, 45 mM NaCl, 1 mM MgSO_4_, 5 mM CaCl_2_, 10 mM dextrose [pH 7.8]) to release spermatocytes and sperm. 20 μl 4% paraformaldehyde (PFA) was added after removal of extra SM buffer, and the slides were kept at room temperature for 15 min. After removal of PFA, 20 μl 4% PFA containing 5% Triton X-100 was added, and the slides were kept for a further 10 min at room temperature. A coverslip was placed on top of the sample, and the slide was frozen on a block that had been cooled in liquid nitrogen while applying gentle pressure to remove the coverslip. The amount of 40 μl PBS (137 mM NaCl, 2.7 mM KCl, 10 mM Na_2_HPO_4_, 1.8 mM KH_2_PO_4_ [pH 7.4]) containing 1% BSA and 10% serum was added to each sample and kept for 1 h to block nonspecific antigens. After removing the blocking buffer, 50 μl fresh primary antibodies (1:200) diluted in blocking buffer was added to each sample and kept overnight at 4°C or 2 h at room temperature. The samples were rinsed 3 times for 10 min each in PBST (PBS containing 0.2% Tween 20), and then were incubated with fluorescently labeled secondary antibodies (1:500) and/or rhodamine-conjugated phalloidin (ThermoFisher R415) (1:10,000) for 45 min in the dark. After 3 rinses with PBST, a drop of mounting medium with DAPI was added to each sample, and a coverslip was placed on top and sealed with nail polish. Images were taken using a spinning-disk confocal microscope.

### Quantification of nucleus-containing residual bodies

Males were dissected in culture medium containing 1 μg/ml Hoechst 33342, which stains nuclei. Nucleus-containing residual bodies were recognized by their larger size, the small and condensed nuclei, and the inhomogeneous cytoplasm. To confirm the numbers of nuclei in each RB, z-stack images were taken and examined. At least 5 worms were dissected, and more than 50 residual bodies were analyzed in each strain.

### Quantification of mitochondria in residual bodies

L4-stage *nmy-2;him-5* and *him-5* males were picked and placed on fresh OP50-seeded plates and grown for 36 h at 25°C. Males were dissected in culture medium containing 50 nM MitoTracker Red and kept at 25°C for at least 30 min. Confocal images were taken, and the number of mitochondria in RB-like structures with attached sperm was quantified. At least 20 RB-like structures were quantified in each strain.

Time-lapse analyses were performed using spermatocytes dissected from *spe-15;him-5* or *him-5* males in culture medium containing 50 nM MitoTracker Red. The number of mitochondria in RBs was quantified at 2 time points: (1) when residual body morphology was clearly seen; (2) when spermatids started to be released from the residual body in *him-5* (approximately 20 min from time point 1), or when spermatids started to retract in *spe-15;him-5* (approximately 15 min from time point 1). At least 20 movies were taken and analyzed for each strain.

### Mating assay

L4-stage *him-5(e1490)* males with rounded tails and L4-stage hermaphrodites from different genetic backgrounds were picked and placed on different fresh OP50-seeded plates for 12 h before mating. The mating assay was carried out by placing 18 celibate *him-5(e1490)* males and 4 celibate hermaphrodites on the same mating plate for 12 h. Each hermaphrodite was transferred to a new plate, and the progeny produced in the first 24 h were quantified.

### Quantification of unfertilized eggs

More than 15 L4-stage hermaphrodites were singled into fresh OP50-seeded plates. Worms were transferred into new plates every 24 h until no eggs were laid. Each time after worms were transferred, the plates were kept for 24 h to allow hatching of the eggs. The progeny were quantified by counting the number of hatched larvae. Unfertilized eggs were stained by adding 5 or 6 drops of 0.025% trypan blue (Amresco k940) onto each plate and were recognized and quantified by their squashy, disc-like shape and their brownish pigmentation because of permeation of the dye. All progeny and unfertilized eggs from the same hermaphrodite were counted, and the percentage of unfertilized eggs was quantified by dividing the total number of unfertilized eggs by the sum of progeny and unfertilized eggs.

### In vitro sperm activation assay

L4-stage males with rounded tails were picked and placed on fresh OP50-seeded plates and grown for 1 to 2 d. The celibate males were then transferred to a new plate without bacteria and allowed to crawl for a few minutes. A slide was marked with a small circle using a PAP pen. About 10 males were dissected in 30 to 50 μL SM buffer containing Pronase E (200 μg/ml) in the circle. After activation for 5 min, a coverslip was gently placed on the surface of the dissected sample, and spermatids or spermatozoa with different morphologies were observed using a 100× oil immersion objective.

### Quantification of fluorescence intensity in sperm

To quantify fluorescence intensity (reporter or immunostaining) in released sperm, images of the same reporter/antibody in the different strains were taken under the same conditions using a 100× objective on a spinning-disk confocal microscope. Background subtraction was performed manually by subtracting the intensity of a background region with the same size as a sperm. The mean intensity of *him-5* sperm was normalized to “1” for comparison. At least 30 sperm were quantified in each strain for RPL-5, actin, and tubulin. At least 15 sperm were quantified for NMY-2.

### Statistical analysis

The SD was used as y-axis error bars for bar charts plotted from the mean value of the data. Data derived from different genetic backgrounds were compared by one-way ANOVA followed by Tukey’s post hoc test as indicated in the figure legends. Data were considered statistically different at *P* < 0.05, *P < 0.05, and **P < 0.0001.

### Plasmid construction

To generate pDBleu-SPE-15(CBD)::FLAG, a cDNA fragment (3091–3660) of the *spe-15 a* isoform was amplified using primers PSYC728/729 and was ligated into pDBleu through *Nhe* I/*Nco* I. To generate pET21b-FLAG::SPE-15(CBD), FLAG::SPE-15(CBD) was amplified from pDBleu-SPE-15(CBD)::FLAG using primers PSYC834/835 and was cloned into pET21b through *Nhe* I/*Hind* III. To introduce mutations (R1067A, R1068A, and L1069A) into pET21b-FLAG::SPE-15(CBD), site-directed mutagenesis was performed using primers PSYC846/847. To construct pET21b-MYC::GIPC-1 and pET21b-MYC::GIPC-2, MYC::GIPC-1 and MYC::GIPC-2 were amplified with PSYC874/838 and PSYC840/841, and then ligated into pET21b through *Nhe* I/*Hind* III. To generate pET21b-SPE-15a (amino acids 838–1219)∷6xHis, a cDNA fragment (2512–3660) of the *spe-15 a* isoform was amplified using primers PHBW399/401 and was ligated into pET21b through *Nde* I/*Hind* III. Primers used for plasmid construction are included in S2 Table.

## Supporting information

S1 Fig Live-cell imaging visualizes the dynamics of actin and tubulin during spermatid differentiation. (A) Time-lapse analysis of meiosis and differentiation in a primary spermatocyte dissected from a *him-5* male. (B) Time required for pseudo-cleavage furrow ingression, RB expansion, and cytokinesis were quantified in *him-5* males with or without SiR-actin/tubulin staining. At least 3 animals were quantified in each condition. Data are shown as mean ± SD. The *him-5* data set without staining was compared with other data sets by one-way ANOVA with Tukey’s post hoc test. Underlying data can be found in S1 Data. (C–E) Time-lapse analysis of meiosis and differentiation in 2 connected secondary spermatocytes that were dissected from *him-5* males expressing both Histone::GFP and Lifeact::RFP (C), stained by SiR-tubulin (D), or expressing both GFP::TBB-2 and mCherry::TBG-1 (E). White arrows in (E) indicate enriched TBB-2 signals, and yellow arrows point to MTOCs labeled by TBG-1. Scale bars: 5 μm. GFP, green fluorescent protein; MTOC, microtubule organizing center; N.S., no significant differences; RB, residual body; RFP, red fluorescent protein; SiR, silicon-rhodamine; TBB-2, tubulin beta 2; TBG-1, gamma tubulin 1.
(TIF)

S2 Fig Myosin II regulates RB formation but not spermatid release. (A) Time-lapse analysis of meiosis and differentiation in 2 connected secondary spermatocytes dissected from *him-5* males expressing NMY-2::GFP and stained by SiR-actin. (B) Time-lapse images of 2 connected secondary spermatocytes dissected from *spe-15(hc75) nmy-2(ne3409);him-5(e1490)* males stained by SiR-actin at the nonpermissive temperature of 25°C. (C) Time-lapse analysis of meiosis in a primary spermatocyte dissected from a *him-5* male expressing GFP::SPE-15. (D) Time-lapse analysis of spermatid release of *him-5* or *nmy-2;him-5* males at the nonpermissive temperature of 25°C. Ten and 15 spermatids out of 30 spermatids were released in *him-5* and *nmy-2;him-5*, respectively. Scale bars: 5 μm. GFP, green fluorescent protein; NMY-2, non-muscle myosin 2; RB, residual body; SiR, silicon-rhodamine; SPE-15, defective spermatogenesis 15.
(TIF)

S3 Fig Loss of myosin VI or GIPC affects segregation of RPL-5, actin, and tubulin into the RB. (A) Time-lapse analysis of meiosis and differentiation in 2 connected secondary spermatocytes dissected from *him-5* males expressing NMY-2::GFP and stained by MitoTracker Red. Double-headed arrow and white lines indicate NMY-2/RB expansion and exclusion of mitochondria from the central region. (B) Fluorescence images of released spermatids from the indicated strains expressing RPL-5::GFP and stained by the DNA label DRAQ5. The relative intensity of RPL-5::GFP was quantified and is shown as mean ± SD. (C) Fluorescence images of released spermatids stained by phalloidin and anti-α-tubulin antibody in the indicated strains. The relative intensities of phalloidin/F-actin and α-tubulin were quantified and are shown as mean ± SD. (D and E) Time-lapse images of meiosis and differentiation in 2 connected secondary spermatocytes dissected from the indicated strains and stained by SiR-tubulin at 20°C (D) or 25°C (E). The arrow points to the microtubules retained in the cortex of spermatids in *spe-15(hc75);him-5(e1490)* animals. (F) Light and fluorescence images of released spermatids from the indicated strains expressing NMY-2::GFP and stained by Hoechst. Relative intensity of NMY-2::GFP in released spermatids was quantified and is shown as mean ± SD. At least 30 and 15 released spermatids were quantified in each strain in (B and C) and (F), respectively. The *him-5* data set was compared with other data sets by one-way ANOVA with Tukey’s post hoc test in each experiment. **P < 0.0001. Underlying data in B, C, and F can be found in S1 Data. Scale bars: 5 μm. DRAQ5, deep red anthraquinone 5; GFP, green fluorescent protein; GIPC, RGS-GAIP-interacting protein C terminus; NMY-2, non-muscle myosin 2; RB, residual body; RPL-5, ribosomal protein 5, large subunit.
(TIF)

S4 Fig Loss of myosin VI but not myosin II affects the final cytokinesis. Time-lapse analysis of meiosis and differentiation in 2 connected secondary spermatocytes that were dissected from *him-5(e1490)* (A), *spe-15(hc75);him-5(e1490)* (B), or *nmy-2(ne3409);him-5(e1490)* (C) expressing both Histone::GFP and Lifeact::RFP at the indicated temperatures. White arrows indicate sites of cytokinesis. Twelve out of 12 spermatids were sealed in *him-5(e1490)* and *nmy-2(ne3409);him-5(e1490)*, whereas 0 out of 12 spermatids were sealed in *spe-15(hc75);him-5(e1490)*. Yellow arrows indicate actin that is retained in spermatids after polarization in *spe-15(hc75);him-5(e1490)*. Scale bars: 5 μm. GFP, green fluorescent protein; RFP, red fluorescent protein.
(TIF)

S5 Fig Loss of myosin VI or GIPC impairs cytokinesis in vivo and causes infertility. (A) DIC and fluorescence images of a nucleus-containing RB that is engulfed and surrounded by CED-1::GFP in *spe-15(hc75);him-5(e1490)* animals expressing CED-1::GFP and mCherry::H2B. (B) DIC and fluorescence images of spermatids and RBs dissected from the indicated strains and stained by Hoechst. The percentage of RBs containing nuclei was quantified and is shown at the right. (C) Quantification of spermatids with different morphologies after pronase treatment in the indicated strains. More than 200 spermatids were examined in each strain. (D) Quantification of self-progeny laid by hermaphrodites of the indicated strains. At least 15 worms were quantified in each strain. (E) Quantification of cross progeny laid in the first 24 h when hermaphrodites of the indicated strains were crossed with *him-5(e1490)* males. Eight hermaphrodites were crossed and quantified in each strain. (F) The percentage of unfertilized eggs laid by hermaphrodites of the indicated strains was quantified. At least 15 worms were quantified in each strain. Data are shown as mean ± SD in (D–F). The *him-5* data set was compared with other data sets by one-way ANOVA with Tukey’s post hoc test in each experiment. **P < 0.0001, *P < 0.05. Underlying data in (B-F) can be found in S1 Data. Scale bars: 5 μm. CED-1, cell death abnormality 1; DIC, differential interference contrast; GFP, green fluorescent protein; GIPC, RGS-GAIP-interacting protein C terminus; N.S., no significant differences; RB, residual body.
(TIF)

S6 Fig Myosin VI associates with actin and GIPC. (A) Time-lapse analysis of meiosis in two connected secondary spermatocytes dissected from *him-5* males expressing GFP::SPE-15 and stained by SiR-actin. The relative intensity of GFP::SPE-15 and SiR-actin along the white line is shown in the graphs. (B) Fluorescence images of spermatids undergoing differentiation dissected from *him-5* animals expressing GFP::SPE-15 and stained by SiR-actin. Yellow arrows indicate colocalized SPE-15 and actin in sperm. White arrows point to colocalized SPE-15 and actin at the sperm–RB boundary. (C) Fluorescence images showing accumulation of GFP::SPE-15 and SiR-actin at the spermatid–RB boundary. (D) Amino acid sequence alignment of GIPC-1 and GIPC-2. Arrows indicate the locations where premature stop codons were introduced into *gipc-1* and *gipc-2* through CRISPR/Cas9. (E) Amino acid sequence alignment of the CBDs of *C*. *elegans* SPE-15 and human myosin VI. The conserved RRL motif is mutated to AAA in the *spe-15(qx529)* mutant by CRISPR/Cas9. (F) Light and fluorescence images of differentiating spermatids dissected from *him-5* males then stained by anti-GIPC and anti-SPE-15 antibodies. White arrows indicate enrichment of GIPC and SPE-15 at the spermatid–RB boundary. Yellow arrows point to faint GIPC and SPE-15 signals at the spermatid poles. The cytosolic staining of SPE-15 is probably caused by recognition of SPE-15 isoforms without the motor domain by the anti-SPE-15 antibody. Scale bars: 5 μm. CBD, cargo-binding domain; CRISPR/Cas9, clustered regularly interspaced short palindromic repeats/CRISPR-associated protein 9 nuclease; GFP, green fluorescent protein; GIPC, RGS-GAIP-interacting protein C terminus; RB, residual body; SiR, silicon-rhodamine; SPE-15, defective spermatogenesis 15.
(TIF)

S1 Video Spermatocytes undergo meiosis and differentiation in vitro. Two connected secondary spermatocytes dissected from *him-5* males were cultured and followed. Time-lapse images were taken every 10 s for 2 h using a DIC microscope and are displayed at 10 frames per second. Selected images are shown in Fig 1B. Scale bar, 5 μm. DIC, differential interference contrast.
(AVI)

S2 Video Mitochondria are excluded from RBs during RB expansion. Two connected secondary spermatocytes dissected from *him-5* males and stained by MitoTracker Red were cultured and followed. Time-lapse images were taken every 10 s for 60 min using a spinning-disk confocal microscope and are displayed at 10 frames per second. Selected and cropped images are shown in Fig 1D. Scale bar, 5 μm. RB, residual body.
(AVI)

S3 Video Ribosomes are fully segregated into the expanding RB. Two connected secondary spermatocytes dissected from *him-5* males expressing RPL-5::GFP were cultured and followed. Time-lapse images were taken every 20 s for 60 min using a spinning-disk confocal microscope and are displayed at 10 frames per second. Selected images are shown in Fig 1E. Scale bar, 5 μm. GFP, green fluorescent protein; RB, residual body; RPL-5, ribosomal protein 5, large subunit.
(AVI)

S4 Video F-actin moves from spermatid poles to the pseudo-cleavage furrow and the expanding RB. Two connected secondary spermatocytes dissected from *him-5* males and stained by SiR-actin were cultured and followed. Time-lapse images were taken every 15 s for 28 min using a spinning-disk confocal microscope and are displayed at 10 frames per second. Selected images are shown in Fig 1F. Scale bar, 5 μm. RB, residual body; SiR, silicon-rhodamine.
(AVI)

S5 Video NMY-2 accumulates at the pseudo-furrow between two undifferentiated spermatids and extends outwards to drive RB expansion. Two connected secondary spermatocytes dissected from *him-5* males expressing NMY-2::GFP were cultured and followed. Time-lapse images were taken every 15 s for 50 min using a spinning-disk confocal microscope and are displayed at 10 frames per second. Selected images are shown in Fig 2A. Scale bar, 5 μm. GFP, green fluorescent protein; NMY-2, non-muscle myosin 2; RB, residual body.
(AVI)

S6 Video Spermatids bud from the remaining cell body in *nmy-2* mutants. Two connected secondary spermatocytes dissected from *nmy-2(ne3409);him-5(e1490)* males were cultured and followed at the nonpermissive temperature of 25°C. Time-lapse images were taken every 10 s for 54 min using a DIC microscope and are displayed at 10 frames per second. Selected images are shown in Fig 2B (lower panels). Scale bar, 5 μm. DIC, differential interference contrast.
(AVI)

S7 Video Haploid spermatids are generated but spermatid differentiation is completely blocked in *spe-15 nmy-2* double mutants. Two connected secondary spermatocytes dissected from *spe-15(hc75) nmy-2(ne3409);him-5(e1490)* males and stained by DRAQ5 were cultured and followed at the nonpermissive temperature (25°C). Time-lapse images were taken every 20 s for 35 min using a spinning-disk confocal microscope and are displayed at 5 frames per second. Selected images are shown in Fig 2E. Scale bar, 5 μm. DRAQ5, deep red anthraquinone 5.
(AVI)

S8 Video SPE-15 moves from spermatid poles to the sperm–RB boundary. Two connected secondary spermatocytes dissected from *him-5* males expressing GFP::SPE-15 were cultured and followed. Time-lapse images were taken every 30 s for 60 min using a spinning-disk confocal microscope and are displayed at 10 frames per second. Selected images are shown in Fig 2F. Scale bar, 5 μm. GFP, green fluorescent protein; RB, residual body; SPE-15, defective spermatogenesis 15.
(AVI)

S9 Video Spermatid budding is concomitant with centripetal movement of SPE-15 and its enrichment at the budding site in *nmy-2* mutants. Two connected secondary spermatocytes dissected from *nmy-2(ne3409);him-5(e1490)* males expressing GFP::SPE-15 were cultured and followed at the nonpermissive temperature of 25°C. Time-lapse images were taken every 20 s for 52 min using a spinning-disk confocal microscope and are displayed at 10 frames per second. Selected images are shown in Fig 2G. Scale bar, 5 μm. GFP, green fluorescent protein; SPE-15, defective spermatogenesis 15.
(AVI)

S10 Video NMY-2 is not restricted to the expanding RB in *spe-15* mutants. Two connected secondary spermatocytes dissected from *spe-15(hc75);him-5(e1490)* males expressing NMY-2::GFP were cultured and followed. Time-lapse images were taken every 1 min for 39 min using a spinning-disk confocal microscope and are displayed at 5 frames per second. Selected images are shown in Fig 2H (top panels). Scale bar, 5 μm. GFP, green fluorescent protein; NMY-2, non-muscle myosin 2; RB, residual body.
(AVI)

S11 Video Mitochondria are not fully segregated into spermatids in *nmy-2* mutants. Two connected secondary spermatocytes dissected from *nmy-2(ne3409);him-5(e1490)* males expressing Histone::GFP and stained by MitoTracker Red were cultured and followed at the nonpermissive 25°C. Time-lapse images were taken every 30 s for 30 min using a spinning-disk confocal microscope and are displayed at 10 frames per second. Selected images are shown in Fig 3A (right panels). Scale bar, 5 μm. GFP, green fluorescent protein.
(AVI)

S12 Video Ribosomes are not fully segregated into the RB in *spe-15* mutants. Two connected secondary spermatocytes dissected from *spe-15(hc75);him-5(e1490)* males expressing RPL-5::GFP were cultured and followed. Time-lapse images were taken every 20 s for 46 min using a spinning-disk confocal microscope and are displayed at 10 frames per second. Selected images are shown in Fig 3D (right panels). Scale bar, 5 μm. GFP, green fluorescent protein; RB, residual body; RPL-5, ribosomal protein 5, large subunit.
(AVI)

S13 Video Cytokinesis is affected in *spe-15* mutants. Two connected secondary spermatocytes dissected from *spe-15(ok153);him-5(e1490)* males were cultured and followed. Time-lapse images were taken every 10 s for 2 h using a DIC microscope and are displayed at 10 frames per second. Selected images are shown in Fig 4A (lower panels). Scale bar, 5 μm. DIC, differential interference contrast.
(AVI)

S14 Video Spatiotemporal enrichment of NMY-2 and SPE-15 during meiosis and spermatid differentiation. Two connected secondary spermatocytes dissected from *him-5(e1490)* males expressing NMY-2::mCherry and GFP::SPE-15 were cultured and followed. Time-lapse images were taken every 20 s for 62 min using a spinning-disk confocal microscope and are displayed at 10 frames per second. Selected images are shown in Fig 4E. Scale bar, 5 μm. GFP, green fluorescent protein; NMY-2, non-muscle myosin 2; SPE-15, defective spermatogenesis 15.
(AVI)

S15 Video Actomyosin VI rings remain when spermatids and RBs are permeabilized by detergent. Two connected secondary spermatocytes dissected from *him-5(e1490)* males expressing GFP::SPE-15 and stained by SiR-actin were cultured and followed. Time-lapse images in 13 z series (0.5 μm/section) were captured every 30 s for 30 min at 20°C using a spinning-disk confocal microscope. NP-40 was added after the first time point. Reconstituted 3D images are displayed at 5 frames per second. Selected DIC and fluorescence images are shown in Fig 5E. Scale bar, 5 μm. DIC, differential interference contrast; GFP, green fluorescent protein; RB, residual body; SiR, silicon-rhodamine; SPE-15, defective spermatogenesis 15.
(AVI)

S1 Data Quantitative observations that underlie the data summarized in Figs 3A and 3B, S1B, S3B, S3C, S3F and S5B–S5F are included. (XLSX)

S1 Table Primers and oligonucleotide templates used for CRISPR/Cas9. CRISPR/Cas9, clustered regularly interspaced short palindromic repeats/CRISPR-associated protein-9 nuclease.
(DOCX)

S2 Table Primers used for plasmid construction. (DOCX)

## Abbreviations

Arp2/3: actin-related proteins 2 and 3
CBD: cargo-binding domain
CED-1: cell death abnormality 1
CRISPR/Cas9: clustered regularly interspaced short palindromic repeats/CRISPR-associated protein 9 nuclease
DIC: differential interference contrast
DRAQ5: deep red anthraquinone 5
FB-MO: Golgi-derived fibrous-body membranous organelle
GFP: green fluorescent protein
GIPC: RGS-GAIP-interacting protein C terminus
H2B: histone 2B
lf: loss of function
LG: linkage group
MTOC: microtubule organizing center
NMY-2: non-muscle myosin 2
PFA: paraformaldehyde
RB: residual body
RFP: red fluorescent protein
RPL-5: ribosomal protein 5, large subunit
SC: synthetic complete
SiR: silicon-rhodamine
SPE-15: defective spermatogenesis 15
TBB-2: beta tubulin 2
TBG-1: gamma tubulin 1
