# Supplementary figures and images for: Distinct roles of two myosins in C. elegans spermatid differentiation
Source: PLoS Biol. 2019 Apr 16;17(4):e3000211. doi: 10.1371/journal.pbio.3000211 (PMC6485759; doi:10.1371/journal.pbio.3000211)

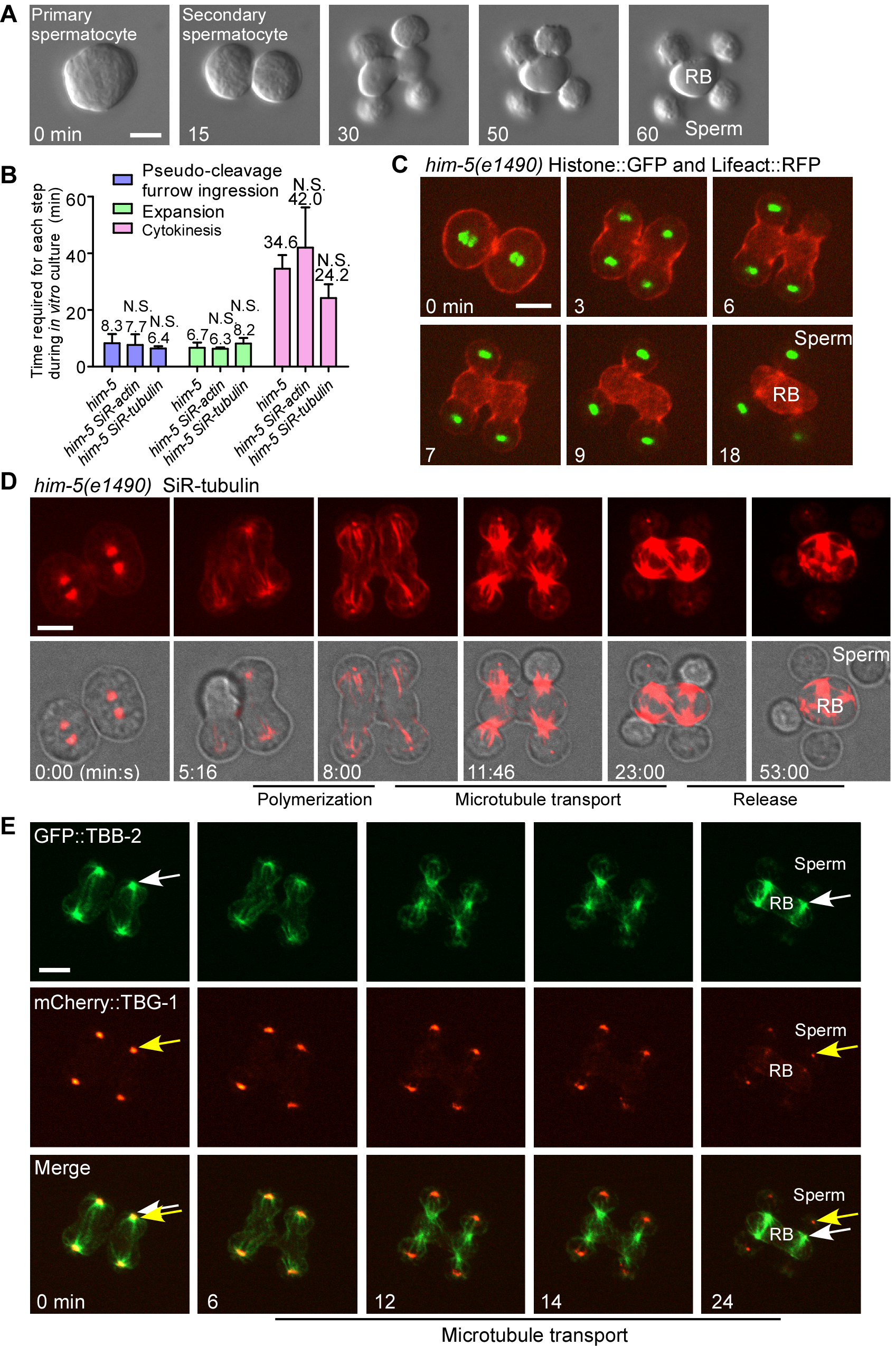

Supplement: S1 Fig — (A) Time-lapse analysis of meiosis and differentiation in a primary spermatocyte dissected from a him-5 male. (B) Time required for pseudo-cleavage furrow ingression, RB expansion, and cytokinesis were quantified in him-5 males with or without SiR-actin/tubulin staining. At least 3 animals were quantified in each condition. Data are shown as mean ± SD. The him-5 data set without staining was compared with other data sets by one-way ANOVA with Tukey’s post hoc test. Underlying data can be found in S1 Data. (C–E) Time-lapse analysis of meiosis and differentiation in 2 connected secondary spermatocytes that were dissected from him-5 males expressing both Histone::GFP and Lifeact::RFP (C), stained by SiR-tubulin (D), or expressing both GFP::TBB-2 and mCherry::TBG-1 (E). White arrows in (E) indicate enriched TBB-2 signals, and yellow arrows point to MTOCs labeled by TBG-1. Scale bars: 5 μm. GFP, green fluorescent protein; MTOC, microtubule organizing center; N.S., no significant differences; RB, residual body; RFP, red fluorescent protein; SiR, silicon-rhodamine; TBB-2, tubulin beta 2; TBG-1, gamma tubulin 1. (TIF) [file pbio.3000211.s001.tif]

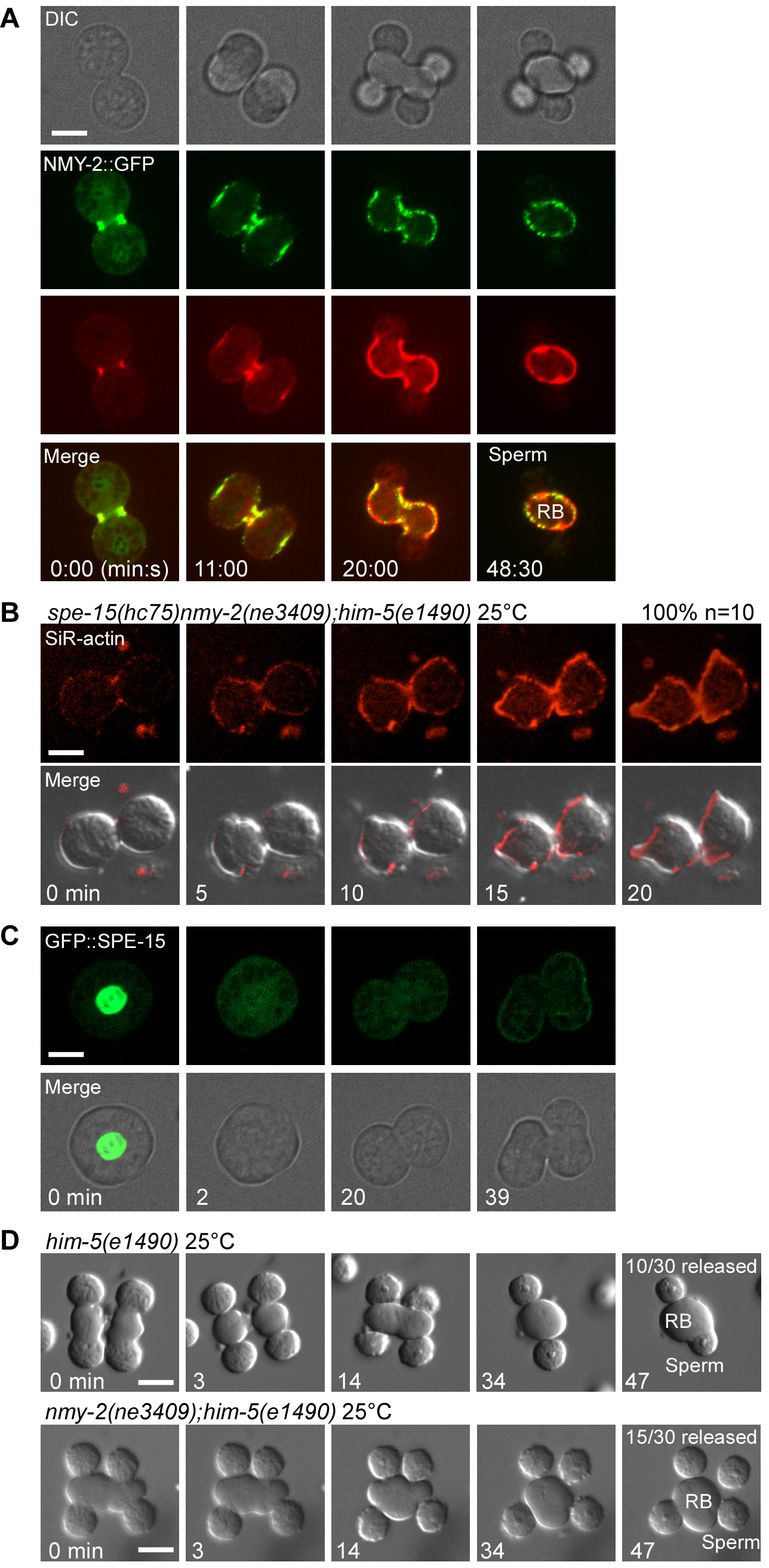

Supplement: S2 Fig — (A) Time-lapse analysis of meiosis and differentiation in 2 connected secondary spermatocytes dissected from him-5 males expressing NMY-2::GFP and stained by SiR-actin. (B) Time-lapse images of 2 connected secondary spermatocytes dissected from spe-15(hc75) nmy-2(ne3409);him-5(e1490) males stained by SiR-actin at the nonpermissive temperature of 25°C. (C) Time-lapse analysis of meiosis in a primary spermatocyte dissected from a him-5 male expressing GFP::SPE-15. (D) Time-lapse analysis of spermatid release of him-5 or nmy-2;him-5 males at the nonpermissive temperature of 25°C. Ten and 15 spermatids out of 30 spermatids were released in him-5 and nmy-2;him-5, respectively. Scale bars: 5 μm. GFP, green fluorescent protein; NMY-2, non-muscle myosin 2; RB, residual body; SiR, silicon-rhodamine; SPE-15, defective spermatogenesis 15. (TIF) [file pbio.3000211.s002.tif]

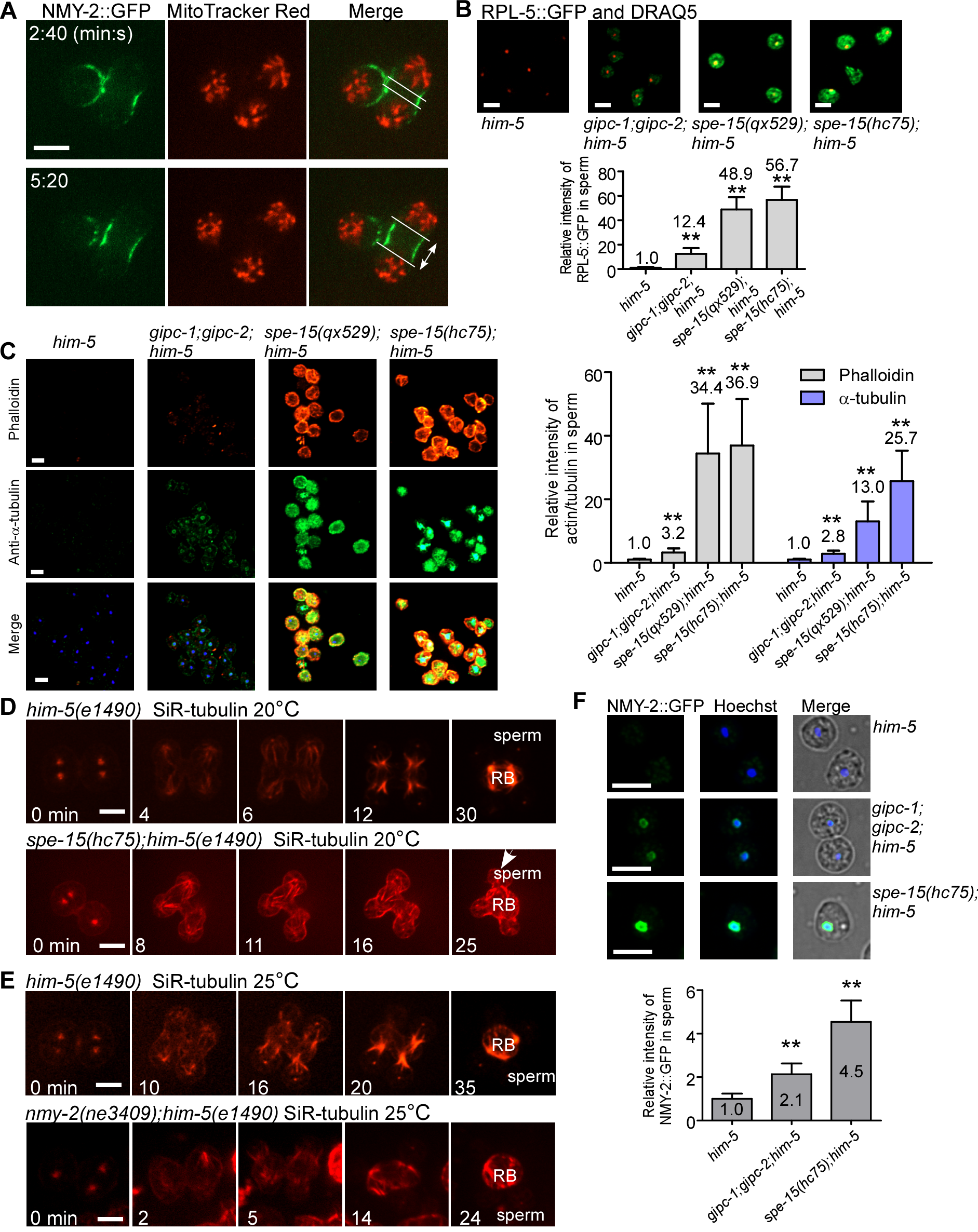

Supplement: S3 Fig — (A) Time-lapse analysis of meiosis and differentiation in 2 connected secondary spermatocytes dissected from him-5 males expressing NMY-2::GFP and stained by MitoTracker Red. Double-headed arrow and white lines indicate NMY-2/RB expansion and exclusion of mitochondria from the central region. (B) Fluorescence images of released spermatids from the indicated strains expressing RPL-5::GFP and stained by the DNA label DRAQ5. The relative intensity of RPL-5::GFP was quantified and is shown as mean ± SD. (C) Fluorescence images of released spermatids stained by phalloidin and anti-α-tubulin antibody in the indicated strains. The relative intensities of phalloidin/F-actin and α-tubulin were quantified and are shown as mean ± SD. (D and E) Time-lapse images of meiosis and differentiation in 2 connected secondary spermatocytes dissected from the indicated strains and stained by SiR-tubulin at 20°C (D) or 25°C (E). The arrow points to the microtubules retained in the cortex of spermatids in spe-15(hc75);him-5(e1490) animals. (F) Light and fluorescence images of released spermatids from the indicated strains expressing NMY-2::GFP and stained by Hoechst. Relative intensity of NMY-2::GFP in released spermatids was quantified and is shown as mean ± SD. At least 30 and 15 released spermatids were quantified in each strain in (B and C) and (F), respectively. The him-5 data set was compared with other data sets by one-way ANOVA with Tukey’s post hoc test in each experiment. **P < 0.0001. Underlying data in B, C, and F can be found in S1 Data. Scale bars: 5 μm. DRAQ5, deep red anthraquinone 5; GFP, green fluorescent protein; GIPC, RGS-GAIP-interacting protein C terminus; NMY-2, non-muscle myosin 2; RB, residual body; RPL-5, ribosomal protein 5, large subunit. (TIF) [file pbio.3000211.s003.tif]

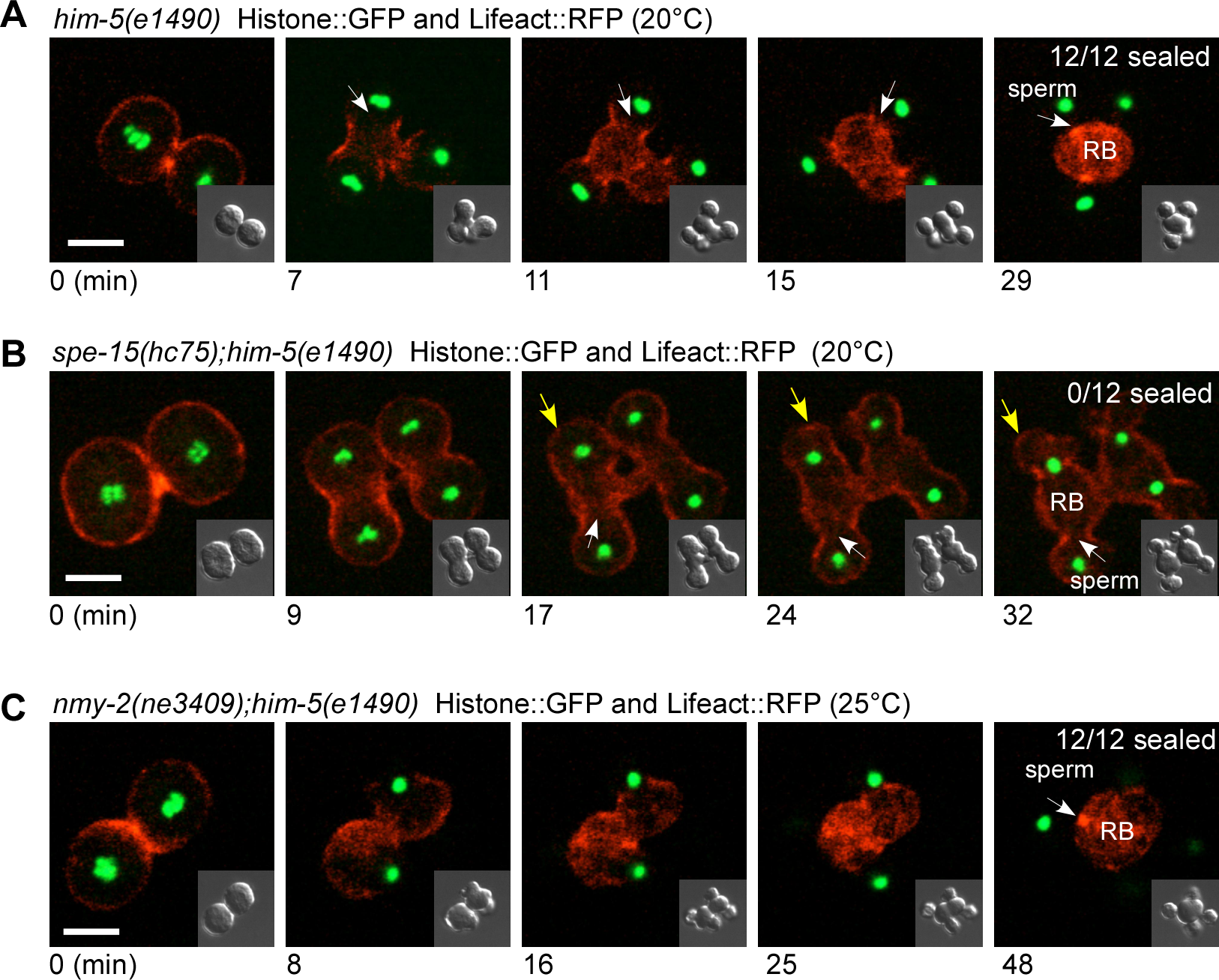

Supplement: S4 Fig — Time-lapse analysis of meiosis and differentiation in 2 connected secondary spermatocytes that were dissected from him-5(e1490) (A), spe-15(hc75);him-5(e1490) (B), or nmy-2(ne3409);him-5(e1490) (C) expressing both Histone::GFP and Lifeact::RFP at the indicated temperatures. White arrows indicate sites of cytokinesis. Twelve out of 12 spermatids were sealed in him-5(e1490) and nmy-2(ne3409);him-5(e1490), whereas 0 out of 12 spermatids were sealed in spe-15(hc75);him-5(e1490). Yellow arrows indicate actin that is retained in spermatids after polarization in spe-15(hc75);him-5(e1490). Scale bars: 5 μm. GFP, green fluorescent protein; RFP, red fluorescent protein. (TIF) [file pbio.3000211.s004.tif]

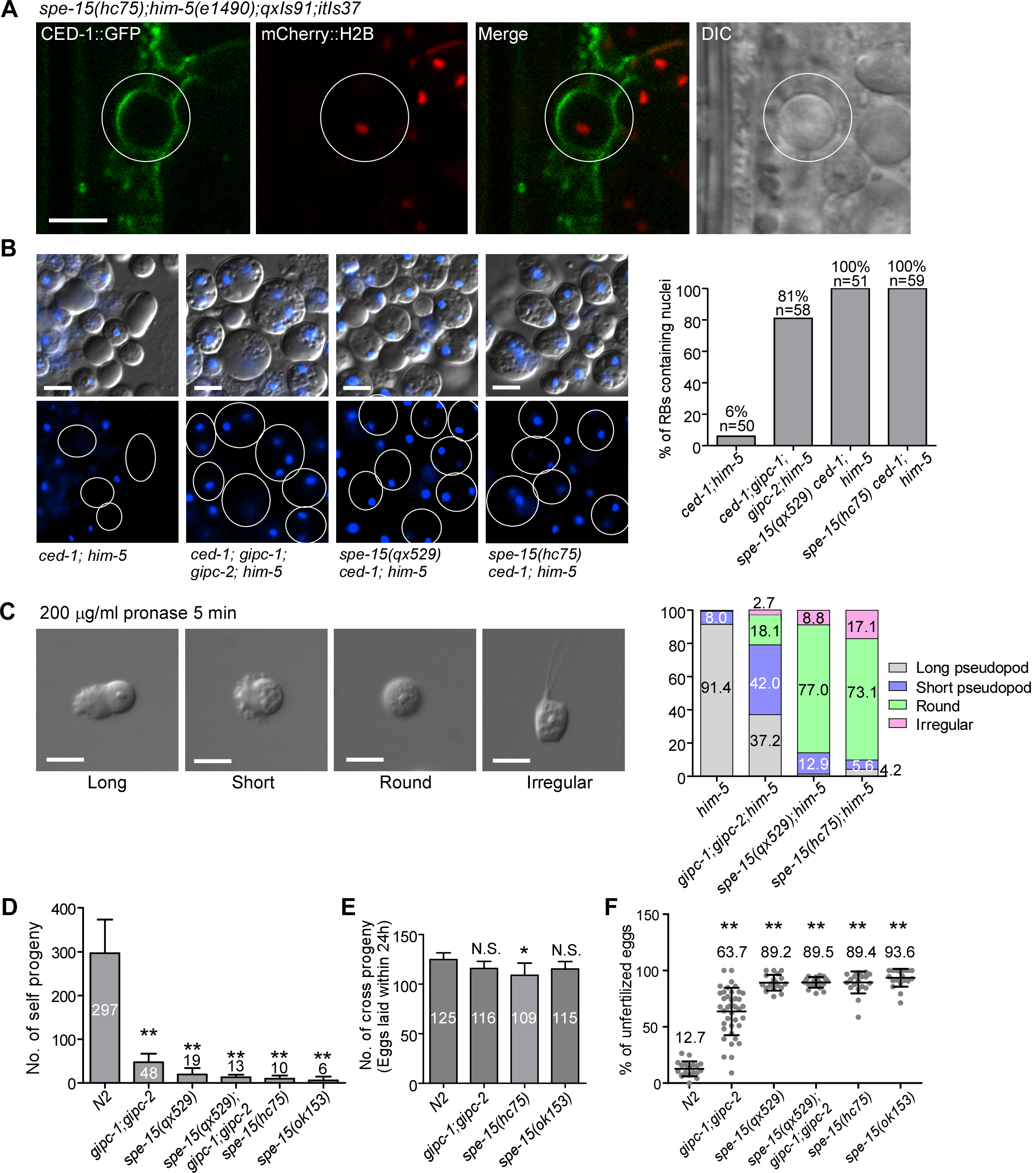

Supplement: S5 Fig — (A) DIC and fluorescence images of a nucleus-containing RB that is engulfed and surrounded by CED-1::GFP in spe-15(hc75);him-5(e1490) animals expressing CED-1::GFP and mCherry::H2B. (B) DIC and fluorescence images of spermatids and RBs dissected from the indicated strains and stained by Hoechst. The percentage of RBs containing nuclei was quantified and is shown at the right. (C) Quantification of spermatids with different morphologies after pronase treatment in the indicated strains. More than 200 spermatids were examined in each strain. (D) Quantification of self-progeny laid by hermaphrodites of the indicated strains. At least 15 worms were quantified in each strain. (E) Quantification of cross progeny laid in the first 24 h when hermaphrodites of the indicated strains were crossed with him-5(e1490) males. Eight hermaphrodites were crossed and quantified in each strain. (F) The percentage of unfertilized eggs laid by hermaphrodites of the indicated strains was quantified. At least 15 worms were quantified in each strain. Data are shown as mean ± SD in (D–F). The him-5 data set was compared with other data sets by one-way ANOVA with Tukey’s post hoc test in each experiment. **P < 0.0001, *P < 0.05. Underlying data in (B-F) can be found in S1 Data. Scale bars: 5 μm. CED-1, cell death abnormality 1; DIC, differential interference contrast; GFP, green fluorescent protein; GIPC, RGS-GAIP-interacting protein C terminus; N.S., no significant differences; RB, residual body. (TIF) [file pbio.3000211.s005.tif]

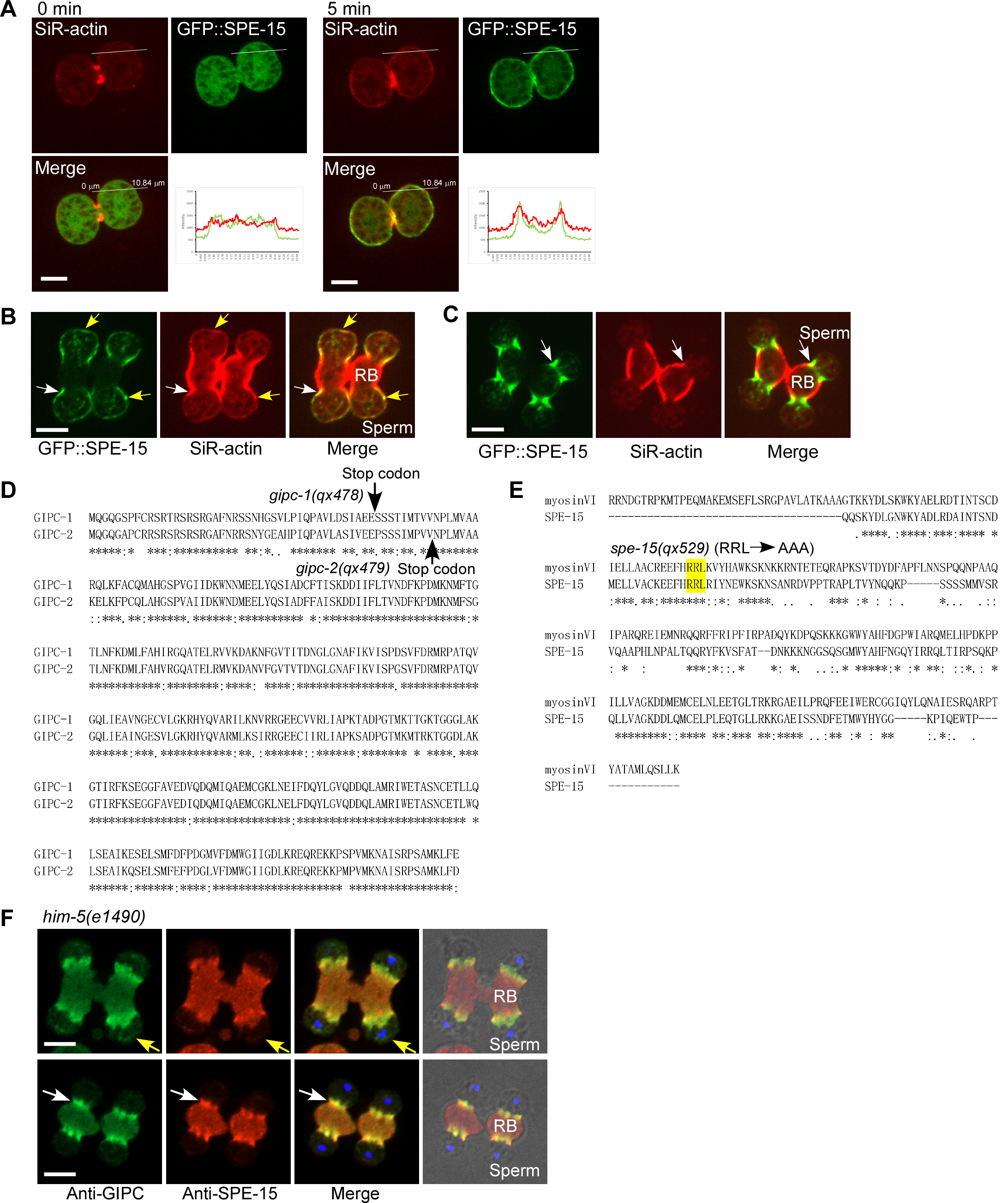

Supplement: S6 Fig — (A) Time-lapse analysis of meiosis in two connected secondary spermatocytes dissected from him-5 males expressing GFP::SPE-15 and stained by SiR-actin. The relative intensity of GFP::SPE-15 and SiR-actin along the white line is shown in the graphs. (B) Fluorescence images of spermatids undergoing differentiation dissected from him-5 animals expressing GFP::SPE-15 and stained by SiR-actin. Yellow arrows indicate colocalized SPE-15 and actin in sperm. White arrows point to colocalized SPE-15 and actin at the sperm–RB boundary. (C) Fluorescence images showing accumulation of GFP::SPE-15 and SiR-actin at the spermatid–RB boundary. (D) Amino acid sequence alignment of GIPC-1 and GIPC-2. Arrows indicate the locations where premature stop codons were introduced into gipc-1 and gipc-2 through CRISPR/Cas9. (E) Amino acid sequence alignment of the CBDs of C. elegans SPE-15 and human myosin VI. The conserved RRL motif is mutated to AAA in the spe-15(qx529) mutant by CRISPR/Cas9. (F) Light and fluorescence images of differentiating spermatids dissected from him-5 males then stained by anti-GIPC and anti-SPE-15 antibodies. White arrows indicate enrichment of GIPC and SPE-15 at the spermatid–RB boundary. Yellow arrows point to faint GIPC and SPE-15 signals at the spermatid poles. The cytosolic staining of SPE-15 is probably caused by recognition of SPE-15 isoforms without the motor domain by the anti-SPE-15 antibody. Scale bars: 5 μm. CBD, cargo-binding domain; CRISPR/Cas9, clustered regularly interspaced short palindromic repeats/CRISPR-associated protein 9 nuclease; GFP, green fluorescent protein; GIPC, RGS-GAIP-interacting protein C terminus; RB, residual body; SiR, silicon-rhodamine; SPE-15, defective spermatogenesis 15. (TIF) [file pbio.3000211.s006.tif]
