# Supplementary material for: Distinct roles of two myosins in C. elegans spermatid differentiation
Source: PLoS Biol. 2019 Apr 16;17(4):e3000211. doi: 10.1371/journal.pbio.3000211 (PMC6485759; doi:10.1371/journal.pbio.3000211)
Supplement: S1 Table — CRISPR/Cas9, clustered regularly interspaced short palindromic repeats/CRISPR-associated protein-9 nuclease. (DOCX) [file pbio.3000211.s023.docx]

S1 Table Primers and oligonucleotide templates used for CRISPR/Cas9

| **Primer** | **Sequence** |
| --- | --- |
| PSL129^1^ | atagtagacgatgattcct GTTTTAGAGCTAGAAATAGCAAGT |
| PSL130^1^ | aggaatcatcgtctactat CAAGACATCTCGCAATAGG |
| PSL139^1^ | ccagtggtgaacccattga GTTTTAGAGCTAGAAATAGCAAGT |
| PSL140^1^ | tcaatgggttcaccactgg CAAGACATCTCGCAATAGG |
| PSYC843^1^ | gtaaatgcgaagacgacga GTTTTAGAGCTAGAAATAGCAAGT |
| PSYC844^1^ | tcgtcgtcttcgcatttac CAAGACATCTCGCAATAGG |
| PJYH59^1^ | ttgcagaaaaaagaagcaa GTTTTAGAGCTAGAAATAGCAAGT |
| PJYH60^1^ | ttgcttcttttttctgcaa CAAGACATCTCGCAATAGG |
| PYBL135^1^ | ctcctgttgctccttgagt GTTTTAGAGCTAGAAATAGCAAGT |
| PYBL136^1^ | actcaaggagcaacaggag CAAGACATCTCGCAATAGG |
| PJYH319^1^ | ttagttgcgaactgagtcg GTTTTAGAGCTAGAAATAGCAAGT |
| PJYH320^1^ | cgactcagttcgcaactaa CAAGACATCTCGCAATAGG |
| PSL131^2^ | ttcagtgcttccaattcagcctgccgtattggattcaattgccgaggaatAATCTAGA gtctactatcatgacagtagtcaatcctttgatggtggcagcaagac |
| PSL141^2^ | gagccaactggcagggaaatttgagctcttttgctgcaaccatcaaCCATGGTTA caccactggcatgattgaagatgatggttcttcgacaattgaagctaaa (Reverse-complement sequence) |
| PSYC845^2^ | gagctcctggtagcctgcaaggaggaattTCATGCTGCTGC tcgcatttacaacgagtggaagtcaaagaactc |
| PJYH61^3^ | aatagagcgtatttgcagaaaaaagaagca ATGAGTAAAGGAGAAGAAC |
| PJYH62^3^ | atccgcggtactatgtgtgctactatccatGGATCC TTTGTATAGTTCATCCATGCC |
| PYBL147^3^ | gaaagcaacgtgttgccgacaagaaggcccttcttctgCAGCTGAAAGAACAGCAAGAG ATGGTGAGCAAGGGCGAGGAGCTG |
| PYBL148^3^ | tacattcatttgaaaacgaaataacaaaaagagagttact TGTACAGCTCGTCCATGCC |
| PJYH428^3^ | caacaccgatgaagaccgcgactcagttcgcaac ATGGTGAGCAAGGGCGAG |
| PJYH429^3^ | caaaaaaataatccggaattaaaaactttagaggtta CTTGTACAGCTCGTCCATGC |

1. Guide sequences are shown in lower case.

2. Mutation regions are shown in upper case. Restriction enzyme recognition sites are underlined. Stop codons are labeled in red.

3. Homology arms at the *spe-15* N-terminus, *rpl-5* C-terminus or *nmy-2* C-terminus are shown in lower case.
