# Supplementary material for: Distinct roles of two myosins in C. elegans spermatid differentiation
Source: PLoS Biol. 2019 Apr 16;17(4):e3000211. doi: 10.1371/journal.pbio.3000211 (PMC6485759; doi:10.1371/journal.pbio.3000211)
Supplement: S2 Table — (DOCX) [file pbio.3000211.s024.docx]

S2 Table Primers used for plasmid construction

| PSYC728^1^ | GG gctagc ATGCAACAGTCGAAGTATGAT |
| --- | --- |
| PSYC729^1^ | GCccatggTTACTTGTCATCGTCATCCTTGTAGTC TGGAGTCCACTCTTGAATTG |
| PSYC834^1^ | GCgctagcGACTACAAGGATGACGATGACAAG CAACAGTCGAAGTATGATTTG |
| PSYC835^1^ | GC aagctt TGGAGTCCACTCTTGAATTGG |
| PSYC846^2^ | GAATTCCATGCTGCTGCTCGCATTTACAACGAGTGGAAGTCAAAGAAC |
| PSYC847^2^ | GTAAATGCGAGCAGCAGCATGGAATTCCTCCTTGCAGGCTACCAGG |
| PSYC874^1^ | GCgctagcGAACAAAAACTCATCTCAGAAGAGGATCTG ATGCAAGGACAAGGATCACCATTC |
| PSYC838^1^ | GC aagctt CTCAAAGAGTTTCATAGCCGATG |
| PSYC840^1^ | GCgctagcGAACAAAAACTCATCTCAGAAGAGGATCTG ATGCAAGGACAAGGAGCCCCATG |
| PSYC841^1^ | GCaagcttGTCAAAGAGTTTCATAGCCGATG |
| PHBW401^1^ | CGGTCAcatatgATG AAAAGACTTGCCATCTTCCGC |
| PHBW399^1^ | CGaagcttTGGAGTCCACTCTTGAATTG |

1. Restriction enzyme sites are shown in lower case.

2. Mutation sites are labeled in red.
